# A self-assembled and H_2_O_2_-activatable hybrid nanoprodrug for lung infection and wound healing therapy

**DOI:** 10.7150/thno.114344

**Published:** 2025-04-28

**Authors:** Rui Zhang, Danhua Mao, Yiyong Fu, Rong Ju, Guoqing Wei

**Affiliations:** Chengdu Women's and Children's Central Hospital, School of Medicine, University of Electronic Science and Technology of China, Chengdu, 611731, China

**Keywords:** Lung infection, Nanoprodrug, Self-assemblies, Synergetic therapy, Wound healing

## Abstract

**Background:** The pursuit of effective antibacterial strategies aimed at mitigating pathogenic bacterial infections while minimising drug resistance remains of paramount importance. A combinational therapeutic strategy that integrates distinct treatment components can enhance overall efficacy and mitigate undesired effects, thereby exhibiting considerable promise in combating bacterial infections.

**Methods:** In this study, a meticulously engineered self-assembling hybrid nanoprodrug (CPBP NPs) has been devised, functioning as a hybrid prodrug of Ciprofloxacin (Cip) and hydroxybenzyl alcohol (HBA).

**Results:** CPBP molecules can generate nanoassemblies via self-assembly and subsequently undergo decomposition to synchronously release Cip and HBA upon hydrogen peroxide (H_2_O_2_) exposure. The CPBP NPs exert antibacterial and anti-inflammatory properties through the controlled release of Cip and HBA, while also facilitating the scavenging of reactive oxygen species. These CPBP NPs exhibit broad-spectrum antibacterial activity against both Gram-negative bacteria (*E. coli*, 98.4%) and Gram-positive bacteria (*S. aureus*, 98.5%). Notably, CPBP NPs not only accumulate in the lungs to facilitate organ-specific infection treatment but also expedite the healing process of infected wounds.

**Conclusions:** Consequently, this H_2_O_2_-activatable hybrid nanoprodrug, possessing excellent biocompatibility, holds substantial promise for advancing clinical applications in managing bacterial infections.

## Introduction

Bacterial infections, including skin wound infections 1^,^ respiratory tract and lung infections 2, and soft tissue infections 3, have emerged as a significant global health concern. Since identifying antibiotics in the 1930s, their utilisation in combating bacterial infections has proven to be a highly effective approach 4. Nevertheless, antibiotic treatment poses inherent risks, including toxicity and the facilitation of bacterial resistance development 5, 6. As a result, developing safe and precisely targeted antibacterial therapies for managing infectious diseases has become an urgent necessity 7, 8. Additionally, a critical aspect that warrants attention is the host immune response upon bacterial invasion. The immune response becomes activated to eradicate the invading pathogens 9, triggering the secretion of pro-inflammatory cytokines encompassing tumour necrosis factor-α (TNF-α), interleukin-1β (IL-1β), interleukin-6 (IL-6), and reactive oxygen species (ROS) by M1-type macrophages 10, 11. However, excessive and dysregulated inflammatory reactions may result in continuous neutrophil migration to infection sites, including the lungs, leading to inflammatory infiltration. This process further induces epithelial and endothelial cell apoptosis, exacerbating tissue damage 12. Consequently, relying solely on anti-infective or immune-suppressing agents proves to be insufficient in treating infectious diseases 13. Instead, a therapeutic strategy combining antibiotics and inflammatory inhibitors has shown potential as an effective intervention. Such a dual-action modality has the potential to simultaneously inhibit bacterial proliferation while modulating inflammation triggered by bacterial infections, thereby mitigating both systemic bacteraemia and excessive immune responses.

At present, with the advancement of nanomedicine, drug nanocarriers have been widely utilised to optimise their therapeutic efficacy while minimising adverse effects on normal tissues or cells 14, 15. Of particular significance is the capability of these nanocarriers to simultaneously encapsulate two or more drugs, thereby facilitating a synergistic approach to disease treatment 16. Conventionally, drugs are incorporated into nanocarriers either through noncovalent interactions or covalent bonding. In the case of the former approach, drugs are associated with nanocarriers via weak hydrophobic forces, π-π stacking interactions, coordination mechanisms, or electrostatic attractions. However, such weak interactions frequently result in premature drug release and unintended cytotoxic effects on non-target cells 17. The issue of early and swift drug discharge can be somewhat addressed through the latter technique, where medicines are chemically bound to nanocarriers 18, 19. This approach leads to the formation of prodrug nanoassemblies, in which prodrugs—covalently modified drug molecules—are capable of releasing their active parent drug *in vivo* via enzymatic and/or chemical conversion 20-22. Nevertheless, the broader therapeutic implementation of prodrug nanoassemblies remains constrained by challenges such as complex synthetic processes, limited scalability in manufacturing, and insufficient drug loading content (DLC).

A hybrid prodrug represents a chemical entity that combines two or more structural components, with each displaying specific biological roles and diverse pharmacological effects 23. These hybrid prodrugs yield multiple distinct pharmacophores, enabling various mechanisms of action to serve as an innovative therapeutic solution 24. Self-assemblies consisting of carefully designed prodrugs have garnered significant interest in drug delivery research in recent years 25, 26. One can accomplish the preparation of self-assembling prodrugs through precise modifications to the chemical composition of parent drugs, including the attachment of hydrophobic or hydrophilic groups through breakable bonds 24, 27. These modified prodrugs possess amphiphilic properties that enable spontaneous assembly in aqueous solutions, creating stable nanostructures capable of liberating therapeutically active compounds when exposed to pathological triggers 28. Self-assembling prodrugs have established a fresh approach to drug delivery, where these compounds offer both structural and therapeutic advantages 29. When juxtaposed with traditional nanoparticulate delivery platforms, prodrug self-assemblies demonstrate notable advantages, such as superior drug loading capacity (reaching 100%), improved dispersion stability, and simplified, economical manufacturing methods 30.

**Scheme 1:**
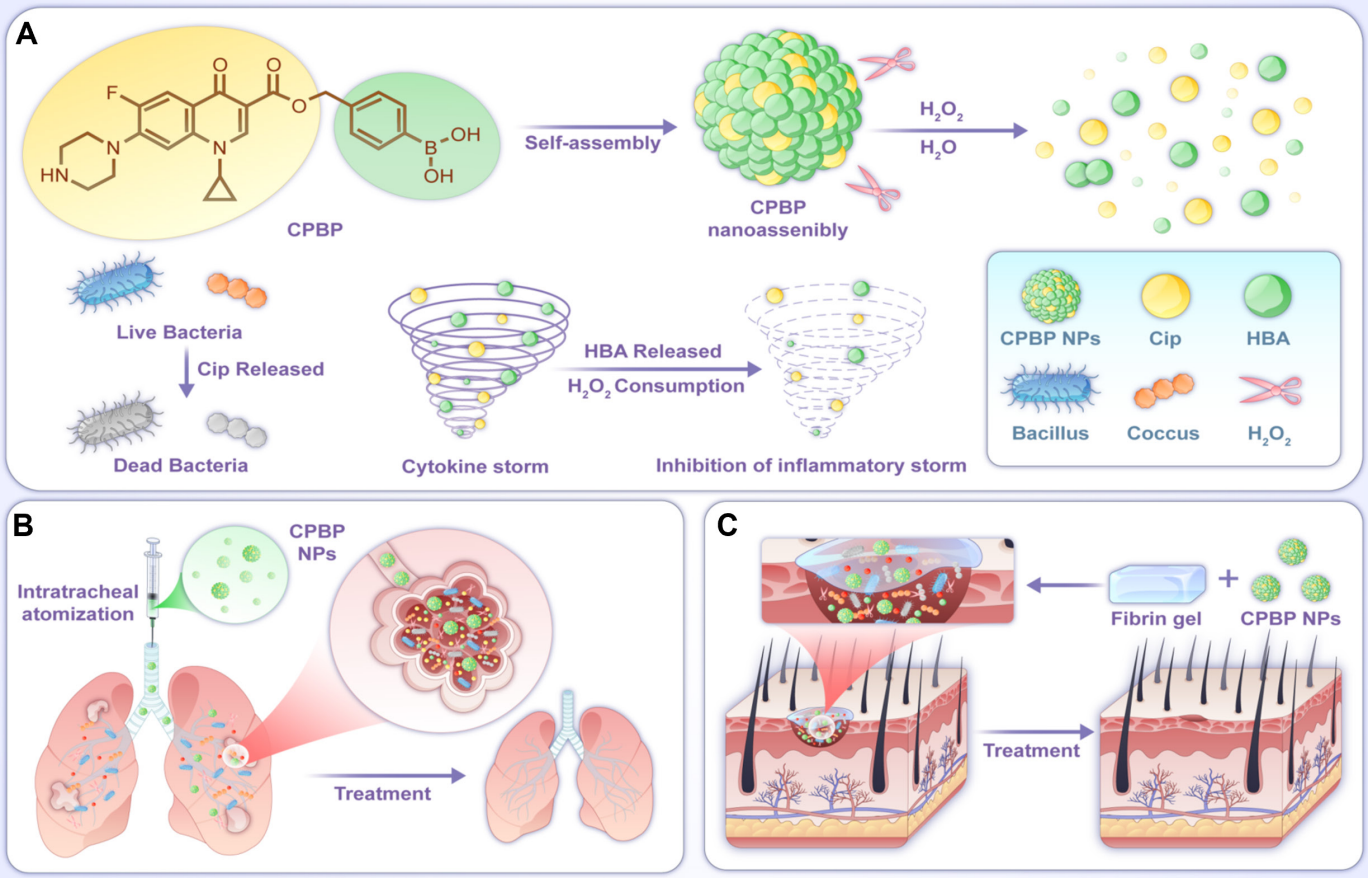
Schematic diagram of the construction and application of CPBP NPs. (A) The preparation process of CBPB NPs and schematic diagram of their antibacterial and anti-inflammatory mechanism. CPBP NPs had an effective therapeutic effect in (B) the lung infection model and (C) the infected skin wound healing model.

By leveraging the advantages of hybrid prodrugs and prodrug self-assembly, a precisely engineered Cip and HBA-based hybrid prodrug (CPBP) was developed, demonstrating the ability to self-assemble into distinct nanostructures, neutralise H₂O₂, and deliver multiple therapeutic outcomes in reaction to pathological triggers linked with infectious diseases (**Scheme 1**). Therefore, CPBP NPs can effectively eliminate ROS while releasing drugs (Cip and HBA) in response to the infected microenvironment. This investigation seeks to clarify the self-assembly process of CPBP, evaluate the translational prospects of CPBP nanoassemblies as a targeted therapeutic approach for infectious diseases (encompassing lung infection and skin wound infection), and examine the underlying mechanism of action.

## Materials and methods

### Materials and agents

Cip, 2,4,6-Trichlorobenzoyl Chloride, 1,4-Dioxane, and p-Hydroxybenzyl Alcohol were obtained from Aladdin (Shanghai, China). 2',7'-dichlorodihydrofluorescein diacetate (DCFH-DA) and thiazolyl blue tetrazolium bromide (MTT) were supplied by Beyotime (Shanghai, China). The Live/Dead BacLight bacterial viability kit was sourced from Thermo Scientific (USA). Dulbecco's Modified Eagle's Medium (DMEM) and fetal bovine serum (FBS) were acquired from Gibco (Grand Island, USA). ELISA kits for mouse TNF-α, interleukin 6 (IL-6), and interleukin 6 (IL-1β) were procured from MultiSciences Biotech Co., Ltd. (Hangzhou, China). All other chemicals, unless stated otherwise, were of analytical grade and were obtained from SinoPharm Chemical Reagent Co., Ltd. (Shanghai, China).

### Cell culture and animals

RAW 264.7 cells were procured from Sichuan University (China), while L-929 and MLE-12 cells were provided by Wuhan Sunncell Biotechnology Co., Ltd. (China). The cells underwent cultivation in DMEM comprising 10% FBS and 1% penicillin-streptomycin (v/v). Cell cultures were maintained in a 5% CO_2_ incubator at 37 °C.

All BALB/c mice (female, aged 6-8 weeks) were acquired from Sichuan Weitong Lihua Experimental Animals Technology Co. Ltd. The animal procedures conducted in this investigation were authorised by the Institutional Animal Care and Use Committee of Sichuan University (Approval Number: 20240826005).

### Preparation and characterisation of CPBP NPs

CPBP NPs were synthesised via a dialysis method. CPBP powder (50 mg) was solubilised in 10 mL of DMSO, and this solution was placed into a dialysis membrane (MWCO 100) and underwent dialysis against deionised water for 3 days to guarantee complete DMSO elimination. The CPBP NPs were then collected through lyophilisation. The morphology and size distribution of the NPs were characterised by utilising SEM and a particle size analyser. The H_2_O_2_ scavenging capacity of CPBP NPs was evaluated using the OxiVision Green probe. A 3.0% H_2_O_2_ stock solution underwent dilution with PBS to generate the H_2_O_2_ solution.

### Antioxidant and anti-inflammatory effects of CPBP NPs

The antioxidant properties of CPBP NPs were assessed by staining H_2_O_2_-activated cells with DCFH-DA. RAW 264.7, L-929, or MLE-12 cells (2 × 10⁵ per dish) cultured in glass bottom dishes were treated with PBS, Cip, HBA, and CPBP NPs. Following a 6-hour incubation, the culture medium was substituted with fresh medium, and the cells were subsequently exposed to 100 μM H_2_O_2_ for 24 h. Afterwards, cells underwent washing with PBS and stained with 5 μM DCFH-DA. Following a 15-minute incubation, the cells underwent gentle washing with PBS before being examined under a fluorescence microscope (FM) (Leica DMi8, Germany).

To evaluate the cytotoxicity and protective capabilities of CPBP NPs, RAW 264.7, L-929, or MLE-12 cells (2 × 10⁵ cells/well) were exposed to stimulation with 200 μM H_2_O_2_ for a duration of 6 h. Following removing the culture medium and adding fresh medium, the cells subsequently received treatments with PBS, Cip, HBA, and CPBP NPs for 24 h. Cell viability was examined utilising the MTT assay per the supplier's protocols. The culture medium from H_2_O_2_-stimulated RAW 264.7 cells was then employed to examine their anti-inflammatory properties. The inflammatory cytokines (TNF-α, IL-1β, and IL-6) levels were quantified employing an ELISA kit.

### *In vitro* antibacterial assays

The antibacterial efficacy of CPBP NPs was assessed utilising the bacteria plate counting method and live/dead staining. *Escherichia coli* (*E. coli*) and *Staphylococcus aureus* (*S. aureus*) underwent cultivation in fresh LB medium on a constant temperature shaker set at 37 °C. The bacterial suspension underwent dilution to reach 10⁶ CFU/mL, and 100 μL of this preparation was transferred into a 96-well plate. Various solution samples (100 μL each) were subsequently added to the plate, with the control cohort receiving only PBS. The 96-well plate was then incubated at 37 °C for 24 h. Following incubation, the bacterial solution was further diluted 10³ times with PBS, and 30 μL of the diluted solution was evenly spread on Luria-Bertani (LB) agar plates. The plates were kept at 37 °C for 12 h before images were captured.

Per the aforementioned procedures, the bacterial precipitate from the co-culture of each cohort was collected. This precipitate was subsequently resuspended in a PBS solution containing SYTO9 and PI, which had been pre-prepared (1 mL of PBS was supplemented with 3 μL PI and 3 μL SYTO9), and kept at 37 °C for 20 min in the dark. After the staining process, bacteria were procured by centrifugation and rinsed thrice with PBS (5,000 rpm, 5 min). A drop of the bacterial suspension was then placed onto a glass slide for sample preparation. The live and dead staining of the bacteria was examined using a FM. Alternatively, the bacterial samples were redispersed in 4% paraformaldehyde and maintained at 37 °C for 1 h. Post-fixation, the specimens underwent dehydration in sequential ethanol concentrations (25%, 50%, 75%, and 100%), each for 10 min. The cell specimens were ultimately resuspended in 100 μL of ethanol for SEM analysis.

### Mouse model of lung infection

The mouse models for lung infection were established per the previously reported methodology with slight modifications 39. Initially, *S. aureus* was cultivated at 37 °C and 200 rpm in LB medium. After approximately 4 h, reaching an OD_600_ = 1, the mice were arbitrarily split into three treatment cohorts (PBS, Cip+HBA, and CPBP NPs). Subsequently, the mice were anesthetised, and each received an intratracheal injection of *S. aureus* at 1 × 10^8^ CFU/mL in 50 μL volume. After 4 and 12 h recovery periods, the mice were administered treatments via intranasal administration (i.n.h.), including PBS, free Cip+ HBA (5 µmol/kg body weight), and CPBP NPs (5 µmol/kg body weight). After 24 h, lung tissue was harvested for H&E staining, and ROS in lung sections were assessed utilising the DCFH-DA probe. ROS production was visualised under a FM. The lungs were then homogenised in 1 mL PBS, serially diluted 10^3^ times, and plated onto LB agar plates. Additionally, the wet/dry weight ratio of the lung tissues was determined, with the wet weight measured before drying and the dry weight after drying. The survival rate of the mice was recorded by checking the animals every 6 h over a total study period of 72 h to evaluate the therapeutic effects.

### Mouse model of infected wound healing

To examine the antibacterial efficacy of CPBP NPs in wound healing, a murine wound model was established based on a previous report 40. Specifically, after anaesthetising the animals, a wound of 5 mm ~ 1 cm in diameter was inflicted on the dorsal region of female BALB/c mice. Following 24 h, the mice were administered a 50 μL injection of *S. aureus* suspension (3 × 10^7^ CFU/mL). The mice were then arbitrarily split into four experimental cohorts: PBS, Cip+HBA, CPBP NPs, and CPBP NPs/G at a dosage of 5 µmol drug /kg body weight. Body weights and wound areas of the mice in all cohorts were monitored throughout the study. After 12 days of treatment, the animals were euthanised, and the wound tissues were procured for histological analysis, including H&E and Masson's staining. Furthermore, immunofluorescence assays for ROS, CD31, CD86, and CD206 were conducted on the wound tissue. Additionally, the wound tissues were swabbed with Q-tips containing sterile saline at day 12, the Q-tips were placed in 1 ml sterile saline respectively, and after ultrasonic shock, serially diluted 10^3^ times, transferred onto LB agar plates and maintained at 37 °C for 24 h.

### *In vivo* immunomodulating ability

The acquired tissue specimens underwent staining procedures with anti-CD86 or anti-CD206 antibodies, succeeded by examination through fluorescence microscopy. Additionally, ELISA was conducted on the collected tissue samples, utilising specific ELISA kits. These encompassed a mouse TNF-α ELISA kit and a mouse IL-6 ELISA kit.

#### *In vitro* and *in vivo* biosafety assessment

Firstly, the cytotoxicity assessment of CPBP NPs was executed using the MTT assay. RAW264.7, L-929, and MLE-12 cells were placed in 96-well plates with 5×10^4^ cells in each well and maintained for 24 h. Subsequently, different doses of CPBP NPs (5, 25, 50, 100, and 200 µg/mL) were introduced into the cell culture medium. Following a 24-hour period, each well received 20 μL of MTT solution (5 mg/mL), and the cells were kept for another 4 h. After this, 100 μL of DMSO was introduced into solubilise the formazan crystals. The absorbance readings at 490 nm were then obtained employing an enzyme-labelled microplate reader (Molecular Devices 1712, USA).

Secondly, fresh blood from BALB/c mice was utilised for the determination of hemolysis. A quantity of 10 mL of blood containing heparin sodium underwent centrifugation at 2,000 rpm for 10 min to remove the serum and isolate the fresh RBCs. The RBCs were then diluted with PBS to prepare a 2% RBC suspension. Following this, 500 μL of the RBC suspension was combined with 1 mL of CPBP NPs at diverse concentrations (25, 50, 100, and 200 µg/mL). Triton X-100 (0.1%) and 0.9% NaCl solution were employed as the positive and negative controls, respectively. Each cohort was kept at 37 °C for 1 h, after which the absorbance of the supernatants was ascertained at 540 nm following centrifugation.

Finally, two cohorts of healthy mice were arbitrarily split into receive treatment with PBS and CPBP NPs via tail vein injection. After 10 days, serum was collected for biochemical index analysis, including BUN, UA, CREA, AST, and ALT. H&E staining was executed on the primary organs, and the daily body weight changes of the mice were tracked throughout the experiment.

### RNA sequencing (RNA-seq) and data analysis

The total RNA was extracted from mouse lung tissues using a RNeasy mini kit (Qiagen), and poly(A) mRNA isolation was performed using Poly(A) mRNA Magnetic Isolation Module (NEB). Library construction and sequencing were performed by GENEWIZ. The quantified RNAs were loaded on an Illumina NovaSeq 6000 instrument using 150 bp paired-end (PE150) configuration. FastQC (version 0.10.1) was used to assess the sequencing quality of the raw data. Trimmomatic was employed to remove the adaptors and trim the raw data to get filter data. Clean reads were then mapped to the mouse GRCm39 genome using Hisat2 (version 2.1.0). Gene expression levels were calculated using Feature Counts (version 1.6.1). Differentially expressed genes (DEGs) were identified using the DESeq2 (version 1.6.3) Bioconductor package. The genes with an adjusted p-value < 0.05 and a fold change > 2 were considered as differentially expressed genes. Gene ontology (GO) enrichment analysis of DEGs was performed by DAVID (https://david.ncifcrf.gov/tools.jsp). Kyoto Encyclopedia of Genes and Genomes (KEGG) analyses were performed using KOBAS (http://bioinfo.org/kobas).

### Statistical analysis

All experiments were executed in triplicate. The date was denoted as means ± standard deviations (S.D.). Statistical significance was determined by unpaired two-tailed Student's t-tests where only two groups existed or by one-way ANOVA for comparison of three or more groups. Differences between groups were considered significant at p-value was below 0.05 (*P < 0.05, **P < 0.01, #, not significant).

## Results and Discussion

### Preparation and characterisations of CPBP nanoassemblies (CPBP NPs)

The CPBP molecule was synthesised through a three-step process, as illustrated in **Figure S1A**. Initially, ciprofloxacin (Cip) was reacted with Boc anhydride, yielding Intermediate 1 (Boc anhydride-containing ciprofloxacin). This intermediate subsequently reacted with phenylboronic acid to produce Intermediate 2. The final CPBP compound was obtained by removing the Boc protecting group from Intermediate 2 using a hydrochloric acid solution. The chemical structure of CPBP underwent confirmation via nuclear magnetic resonance (NMR) and mass spectrometry (MS) analyses (**Figure S2-S4**). Furthermore, the Fourier transform infrared (FT-IR) and ultraviolet-visible (UV-vis) spectra of the CPBP molecule are provided in **Figures S5-S6**.

**Figure 1:**
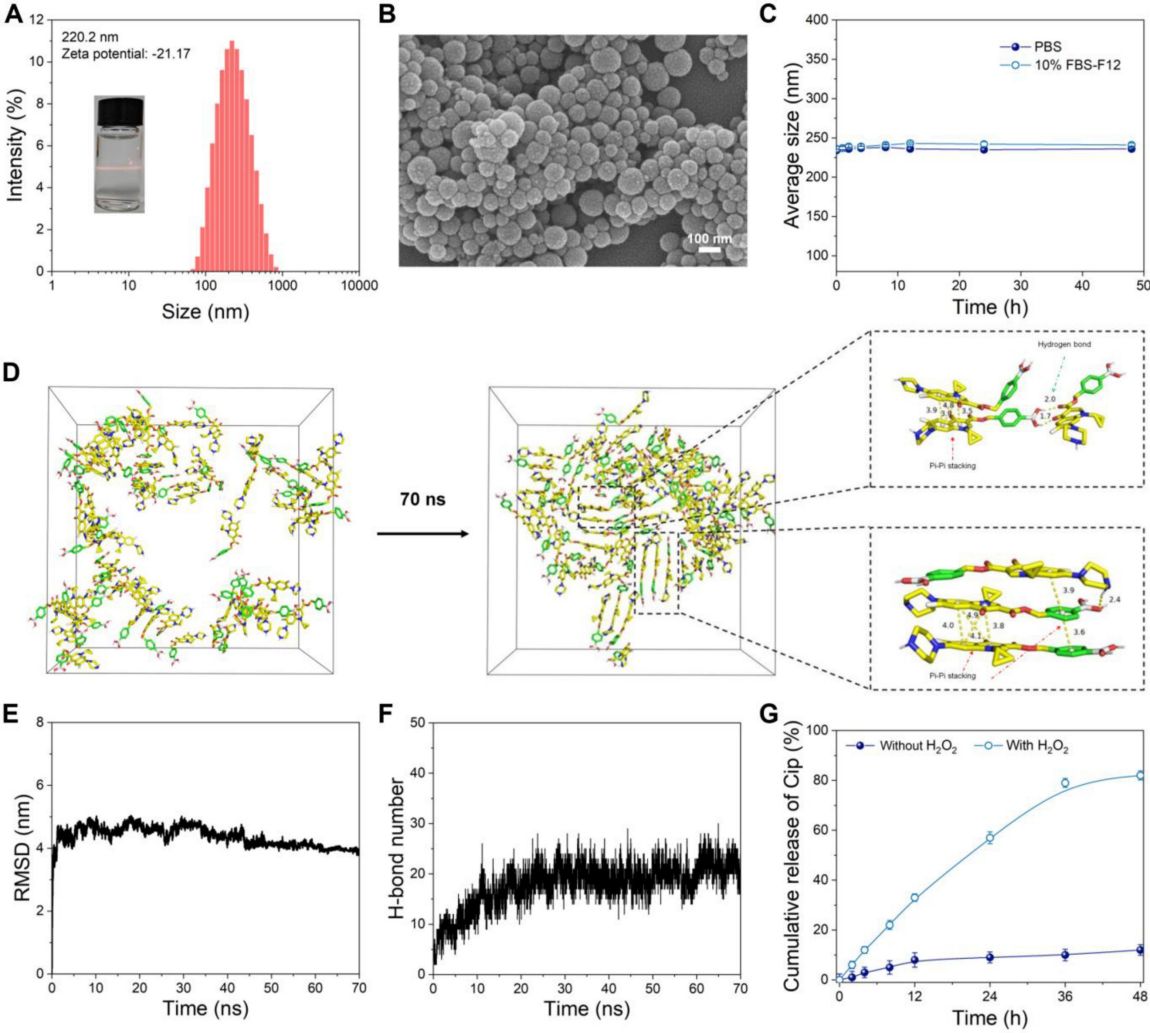
Characterization of CPBP NPs. (A) Size distribution histogram and Tindall effect of CPBP NPs solution. (B) SEM image of CPBP NPs. (C) DLS results regarding the size of CPBP NPs for 48 h in PBS and 10% FBS-F12 medium (n = 3). (D) Structural changes of initial CPBP molecules in the system during simulation and intermolecular interaction patterns in the CPBP system. (E) The RMSD changes of CPBP molecules in the system during simulation. (F) The total number of formed hydrogen bonds between CPBP molecules in the system during the self-assembly process. (G) Cumulative release of Cip from CPBP NPs (n = 3).

The CPBP nanoassembly was formed via the self-assembly of CPBP molecules in an aqueous solution. A distinct Tyndall light-scattering effect was observed in the CPBP NPs solution (**Figure 1A**). Dynamic light scattering (DLS) analysis suggested that CPBP NPs exhibited an average particle dimension of 220.2 ± 5.75 nm, whilst demonstrating a polydispersity index (PDI = Mw/Mn) of 0.19 ± 0.06. Furthermore, the surface charge of the CPBP NPs solution was determined to be -21.17 ± 0.76 mV. Scanning electron microscopy (SEM) revealed that CPBP NPs exhibited a well-defined and uniform nanomorphology (**Figure 1B**). To assess the stability of CPBP NPs, measurements were conducted in a 10% FBS-F12 medium solution and phosphate buffer solution (PBS). The findings demonstrated that the hydrodynamic size of the nanoparticles remained unchanged over 48 h, confirming their stability and suitability for further experimentation (**Figure 1C**). To gain deeper insights into the self-assembly process of CPBP NPs, molecular dynamics simulations were performed. As depicted in **Figure 3D**, CPBP molecules rapidly self-assembled into nanoparticles within a remarkably short timeframe (70 ns). Throughout the assembly process, the total energy and intermolecular forces exhibited a gradual decline before reaching equilibrium (**Figure 3E**). Meanwhile, the benzene rings within CPBP molecules established a π-π stacking configuration, accompanied by hydrogen bond formation. Additionally, the number of hydrogen bonds progressively increased and eventually stabilised (**Figure 3F**). These findings prove that CPBP nanosizing occurs with exceptional rapidity under experimental conditions, with intermolecular hydrogen bonding and π-π stacking playing a dominant role in nanoparticle formation.

**Figure 2:**
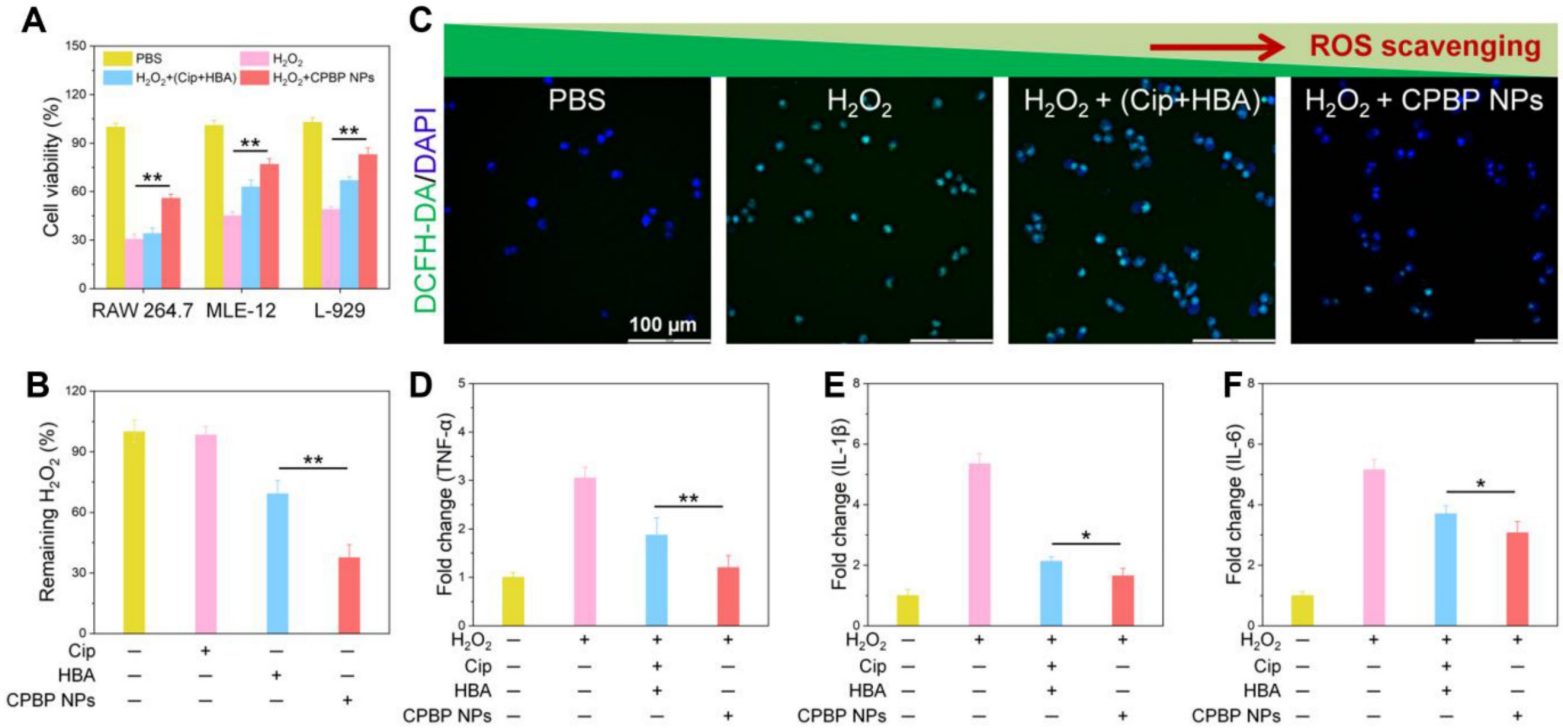
Antioxidant and anti-inflammatory activity of CPBP NPs. (A) Protective effects CPBP NPs on H_2_O_2_-stimulated RAW 264.7, MLE-12, and L-929 cells (n = 3), **P < 0.01. After 3 h of treatment with H_2_O_2_ (200 μM), cells were incubated with Cip+HBA or CPBP NPs. (B) OxiVision Green assay for determining the amount of H_2_O_2_ eliminated by adding Cip+HBA or CPBP NPs (n = 3), **P < 0.01. (C) Suppressive effects of Cip+HBA or CPBP NPs on the generation of intracellular ROS in H_2_O_2_-stimulated cells, DAPI: excitation wavelengths is 405 nm, DCFH-DA: excitation wavelengths is 488 nm. Suppressive effects on Cip+HBA or CPBP NPs on the expression of TNF-α (D), IL-1β (E), and IL-6 (F) in H_2_O_2_-stimulated cells (n = 3), **P < 0.01 and *P < 0.05.

To assess the H₂O₂-responsiveness of CPBP NPs (**Figure S1B**), the disintegration of nanoassemblies was first investigated utilising SEM. As demonstrated in **Figure S7**, the spherical configuration of CPBP NPs was no longer observed following incubation in H₂O₂ for 12 h. Subsequently, the release profiles of Cip and HBA during their H₂O₂-induced degradation were further analysed. As illustrated in **Figure 1G**, CPBP NPs demonstrated a H₂O₂-dependent release behaviour for Cip. A substantial proportion of Cip was released within the first 12 h in the presence of H₂O₂, whereas approximately 20% was released over 48 h in the absence of H₂O₂. Additionally, findings revealed that the quantity of HBA liberated through H₂O₂-induced degradation of CPBP NPs reached approximately 80 wt% within 48 h (**Figure S8**). Moreover, the drug release triggered by H₂O₂ was further validated through high-performance liquid chromatography (HPLC) analysis (**Figure S9**). Given that CPBP NPs was designed to undergo H₂O₂-induced degradation to facilitate drug release while simultaneously consuming H₂O₂, its H₂O₂-scavenging capacity was investigated using a H₂O₂ fluorescent probe (**Figure 2A**). It was observed that 100 μg/mL of CPBP NPs effectively eliminated the majority of H₂O₂, whereas an equivalent amount of Cip (21 μM) and HBA (21 μM) alone exhibited no detectable scavenging activity.

### Antioxidant and anti-inflammatory activities of CPBP NPs

In experiments utilising RAW 264.7, L-929, and MLE-12 cell lines, researchers evaluated the antioxidant capabilities of CPBP NPs at 100 μg/mL, equivalent to 21 μM Cip and 21 μM HBA. Exposure to 100 μM H₂O₂ led to a considerable decrease in cell viability, aligning with previous research findings 33, 34. While Cip exhibited minimal impact on cell viability, HBA markedly enhanced cell survival, attributed to its strong antioxidant capabilities, including free radical scavenging and inhibiting lipid peroxidation and protein oxidation 35. As anticipated, CPBP NPs effectively mitigated H₂O₂-induced cytotoxicity across all cell lines (**Figure 2B**). Notably, CPBP NPs at 50 μg/mL demonstrated markedly greater protective effects compared to an equivalent combination of Cip and HBA. The enhanced antioxidant activity of CPBP NPs is attributed to the synergistic effect of CPBP's H₂O₂-scavenging ability and the inherent antioxidant properties of HBA.

To examine the antioxidant capabilities of CPBP NPs, the cells underwent staining with DCFH-DA, a ROS probe and were subsequently examined using fluorescence microscopy. Cells stimulated with H₂O₂ generated a substantial amount of ROS, as indicated by the presence of intense green fluorescence. Although cotreatment with Cip and HBA reduced intracellular ROS levels, the effect was not statistically significant. Conversely, exposure to 100 μg/mL of CPBP NPs resulted in the near-complete suppression of H₂O₂-induced ROS generation (**Figure 2C and S10**). These findings provide strong evidence that CPBP NPs effectively scavenge H₂O₂ and exhibit potent antioxidant properties by inhibiting intracellular ROS.

To assess whether CPBP NPs suppress pro-inflammatory cytokine levels, RAW 264.7 cells were exposed to CPBP NPs (100 μg/mL) and subsequently stimulated with H₂O₂. The stimulation with H₂O₂ led to a substantial elevation of pro-inflammatory cytokines, including TNF-α, IL-1β, and IL-6 (**Figure 2D-F**). Both Cip and HBA exhibited moderate inhibitory effects on the expression of these cytokines. However, CPBP NPs substantially reduced TNF-α, IL-1β, and IL-6 levels, demonstrating a markedly stronger inhibitory effect than the equivalent combination of Cip and HBA.

### *In vitro* antibacterial test of CPBP NPs

**Figure 3:**
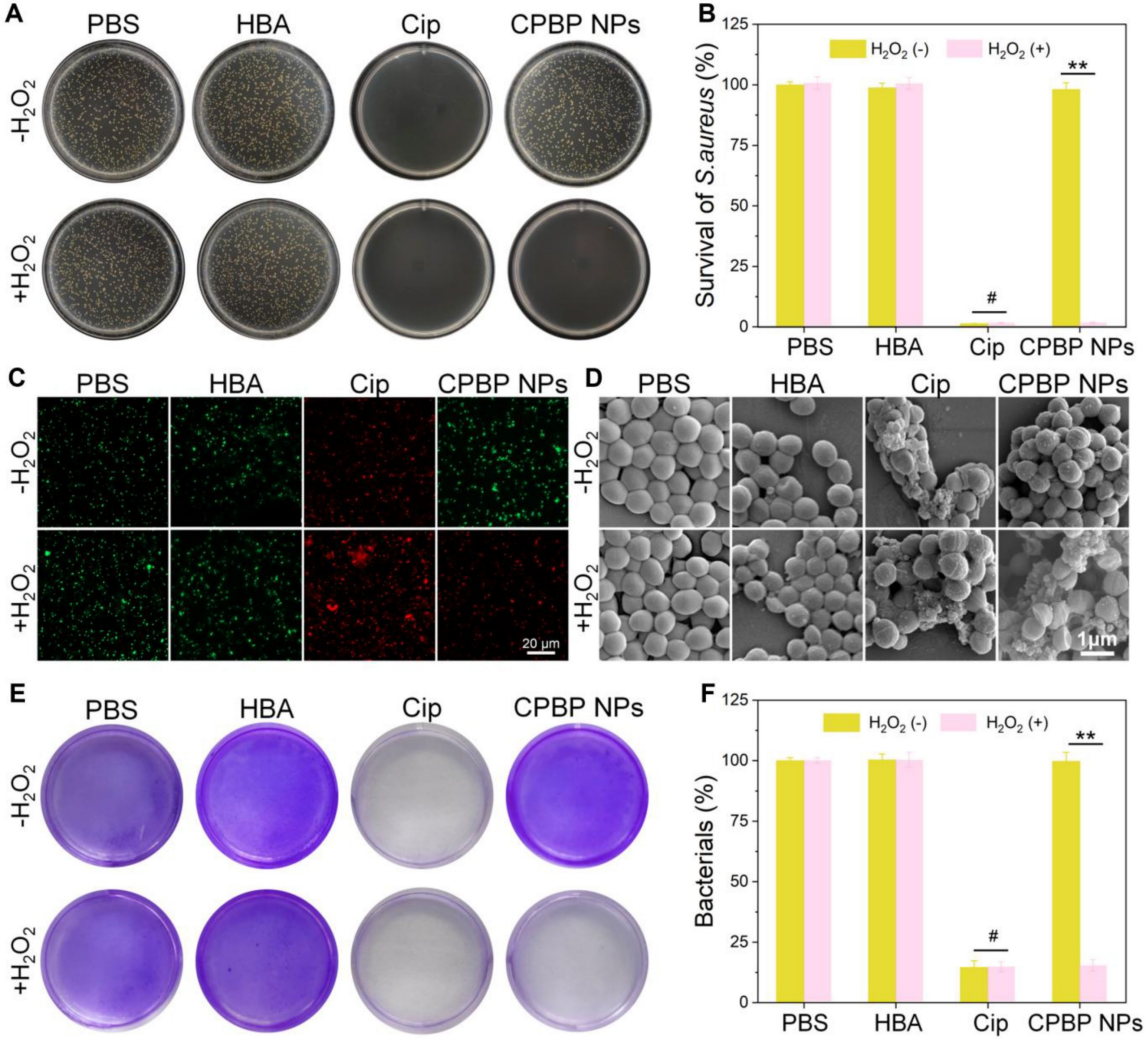
Antibacterial activity of CPBP NPs. (A) Representative photographs and (B) their quantitative analysis of bacterial colonies formed on agar plates in different treatment groups (n = 3), *P* < 0.01. ^#^*P* > 0.05. (C) Fluorescence images for *S. aureus* after various treatments, green and red represent live and dead bacteria respectively. (D) SEM images of *S. aureus* after various treatments. E) Biofilm biomass of *S. aureus* identified by crystal violet staining after various treatments and (F) the quantitative evaluation of biofilm clearance ratio (n = 3), **P < 0.01. ^#^*P* > 0.05.

The antibacterial efficacy of CPBP NPs against Gram-positive bacteria (*S. aureus*) and Gram-negative bacteria (*E. coli*) was assessed *in vitro* employing the bacterial plate counting method. PBS was employed as the control cohort. As illustrated in **Figure 3A, B** and **Figure S11A, B**, considerable bacterial colony formation was observed on LB agar plates within both control and HBA cohorts, irrespective of H₂O₂ presence in the medium, suggesting that HBA had no inhibitory effect on the growth of *S. aureus* and *E. coli*. As anticipated, following exposure to CPBP NPs, a marked reduction in the number of *S. aureus* and *E. coli* colonies was observed in the H₂O₂-containing cohort. The bacterial survival rates of *S. aureus* and *E. coli* declined to 1.5% and 1.6%, respectively, which were comparable to those recorded in the Cip-treated cohorts.

To further elucidate the antibacterial activity *in vitro*, a live/dead bacterial cell staining assay was conducted to assess antibacterial efficacy. Live bacteria were labelled with SYTO9, emitting green fluorescence, while dead bacteria were stained with PI, displaying red fluorescence. As depicted in **Figure 3C** and **Figure S11C**, *S. aureus* and* E. coli* in the PBS and HBA treatment cohorts exhibited green fluorescence with minimal red fluorescence, suggesting that HBA had a negligible impact on bacterial viability. A similar pattern was observed in the CPBP NPs treatment cohort. Conversely, in the presence of H₂O₂, the CPBP NPs-treated cohort displayed intense red fluorescence, confirming that H₂O₂ facilitated the release of Cip.

Subsequently, bacterial morphology following various treatments was examined using SEM. In the acquired images, yellow arrows indicate evident membrane damage and intracellular content leakage. As depicted in *S. aureus* (**Figure 3D**) and *E. coli* (**Figure S11D**) analyses, bacterial surfaces in the PBS, HBA, and CPBP NPs cohorts remained smooth and intact. However, after co-incubation with free Cip, both *S. aureus* and *E. coli* exhibited severe structural damage, leading to the complete loss of normal cell morphology. In the CPBP NPs treatment cohort, bacterial cells displayed slight membrane folding and leakage, implying that CPBP NPs induced minimal bacterial damage. Conversely, upon exposure to CPBP NPs+H₂O₂, *S. aureus* and *E. coli* exhibited pronounced membrane deformation and leakage, suggesting that the antibacterial potency of CPBP NPs is markedly enhanced upon activation by H₂O₂.

In an infected environment, bacteria evade the immune system and external bactericidal agents by secreting extracellular polymeric substances to establish biofilms, contributing to the persistent and treatment-resistant nature of biofilm-associated infections 36, 37. Consequently, *S. aureus* was selected as a representative bacterium to evaluate the efficacy of CPBP NPs in eradicating biofilm infections using crystal violet staining. As illustrated in **Figure 3E**, biofilms maintained high structural integrity following PBS and HBA treatments, suggesting that the dense biofilm matrix remained largely unaffected by HBA. In contrast, biofilms treated with Cip or CPBP NPs in combination with H₂O₂ exhibited significant structural disruption. These findings indicate that CPBP NPs exert biofilm-disrupting effects through an H₂O₂ activation mechanism. Quantitative analysis of crystal violet staining confirmed that CPBP NPs combined with H₂O₂ resulted in the lowest biofilm biomass, comparable to that observed in the Cip-treated cohort (**Figure 3F**). In summary, H₂O₂ activation enables CPBP NPs to degrade biofilms and effectively eliminate bacteria.

### Antibacterial activity of CPBP NPs in lung infection

**Figure 4:**
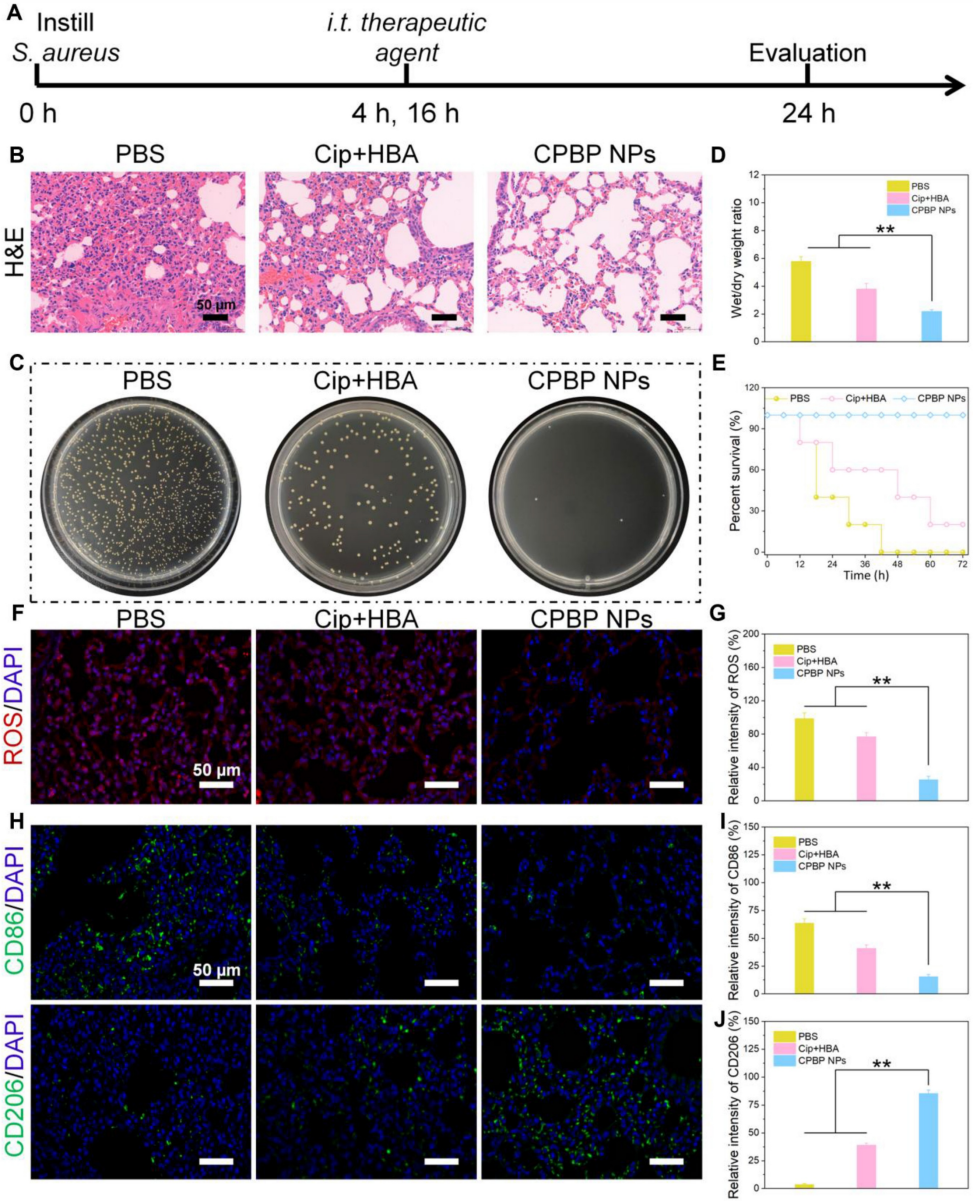
Therapeutic effect on lung infection with *S. aureus*. (A) Schematic diagram of lung infection treatment schedule in PBS, Cip+HBA, and CPBP NPs. (B) Representative images of lung sections H&E with various treatments. (C) Photographs of bacterial colonies obtained from infected lung tissues. (D) Wet/dry weight ratio of lungs (n= 5), **P < 0.01. (E) Survival curves of mice with bacterial pneumonia in various treatment groups (n = 8). (F) Immunofluorescence staining for ROS by DCFH-DA fluorescence probe (red is a false color) and (G) their relative intensity, **P < 0.01. (H) Immunofluorescence staining images of CD86 and CD206 in lung infection of mice after various treatments and their relative intensity of CD86 (I) and CD206 (J), **P < 0.01.

After evaluating the antibacterial effects of CPBP NPs *in vitro*, their antibacterial efficacy was further investigated in a murine lung infection model to validate the previous inference. The experimental design is outlined in **Figure 4A**. In brief, female BALB/c mice were anaesthetised and subsequently administered *S. aureus* (1 × 10⁸ CFU/mL, 50 μL) via intratracheal instillation. At 4 and 16 h post-infection, the mice were treated by intratracheal injection of PBS, Cip+HBA, or CPBP NPs. Upon completion of the treatment regimen, mice were euthanised, and lung tissues were harvested for further analysis. Histological examination through hematoxylin and eosin (H&E) staining revealed that the alveolar septa in the PBS-treated cohort were markedly thickened, accompanied by pronounced infiltration of macrophages and neutrophils, confirming the successful establishment of the lung infection model (**Figure 4B**). Severe tissue damage and extensive inflammatory cell infiltration were observed in both the PBS and Cip+HBA cohorts. However, in contrast to other treatment cohorts, lung tissues from the CPBP NPs-treated cohort exhibited notably improved alveolar integrity, with a markedly lower number of inflammatory cells, clearly demonstrating that inflammation was alleviated and lung tissue damage was effectively repaired.

Additionally, the residual bacterial load in lung tissues was assessed using the spread plate method. A considerable decrease in bacterial count was detected in the lungs of mice administered with CPBP NPs. However, substantial bacterial presence was detected in the lung tissues of other treatment cohorts, suggesting that CPBP NPs exhibited potent antibacterial activity within the lungs (**Figure 4C**). Furthermore, bacterial infections in the lungs can trigger excessive inflammatory secretions, leading to pulmonary oedema. Thus, the severity of pulmonary oedema was evaluated by determining the wet/dry weight ratio of lung tissues. A comparison between lung tissues from the CPBP NPs-treated cohort and those from the PBS and Cip+HBA cohorts revealed a markedly lower wet/dry ratio, indicating effective alleviation of pulmonary oedema (**Figure 4D**). In addition, the survival rate of mice subjected to different treatments was monitored. In the Cip+HBA cohort, only 20% of the mice survived, whereas the survival rate reached 100% in the CPBP NPs-treated cohort, demonstrating that the administration of CPBP NPs substantially enhanced survival outcomes in mice afflicted with bacterial pneumonia (**Figure 4E**). Subsequently, DCFH-DA staining was employed as a fluorescent probe to investigate ROS generation *in vivo*. The weakest red fluorescence signal was detected in mice that received intratracheal instillation of CPBP NPs, in contrast to the PBS and Cip+HBA cohorts, which could be explained by the enzymatic transformation of endogenous H₂O₂ (**Figure 4F and G**).

Macrophages serve a pivotal function in regulating inflammatory responses and facilitating tissue repair. M1-like macrophages function as pro-inflammatory immune cells, contributing to the inflammatory process by releasing inflammatory mediators in the acute infection phase, while M2-like macrophages exhibit anti-inflammatory properties and support tissue regeneration 38. To elucidate the antibacterial efficacy of CPBP NPs in bacterial pneumonia, the proportion of different macrophage subtypes in lung tissues following various treatments was assessed through immunohistochemistry (IHC). The IHC findings revealed that severe infection was evident in both the PBS and Cip+HBA cohorts, accompanied by a marked elevation in the percentage of M1-like macrophages (CD86^+^). In contrast, the CPBP NPs-treated cohort exhibited a higher infiltration of M2-like macrophages (CD206^+^) with a concurrent reduction in M1-like macrophages in the lung tissues (**Figure 4H-J**). These findings suggest that CPBP NPs treatment effectively mitigated infection-induced immune responses and alleviated local inflammatory symptoms, thereby facilitating the remodelling of inflammation-associated macrophages in lung tissues.

Infection with *S. aureus* resulted in a considerable elevation in inflammatory cytokine expression, specifically TNF-αand IL-6. Consequently, lung tissue and serum specimens were procured 48 h post-treatment administration, and the cytokine concentrations were ascertained utilising enzyme-linked immunosorbent assay (ELISA) (**Figure S12**). In the PBS cohort, TNF-α and IL-6 exhibited markedly high expression levels. In contrast, inflammation was alleviated to varying degrees following different treatments, with CPBP NPs leading to the lowest cytokine expression levels, indicating its enhanced anti-inflammatory efficacy.

### Transcriptomic Analysis after CPBP NPs treatment in lung infection

**Figure 5:**
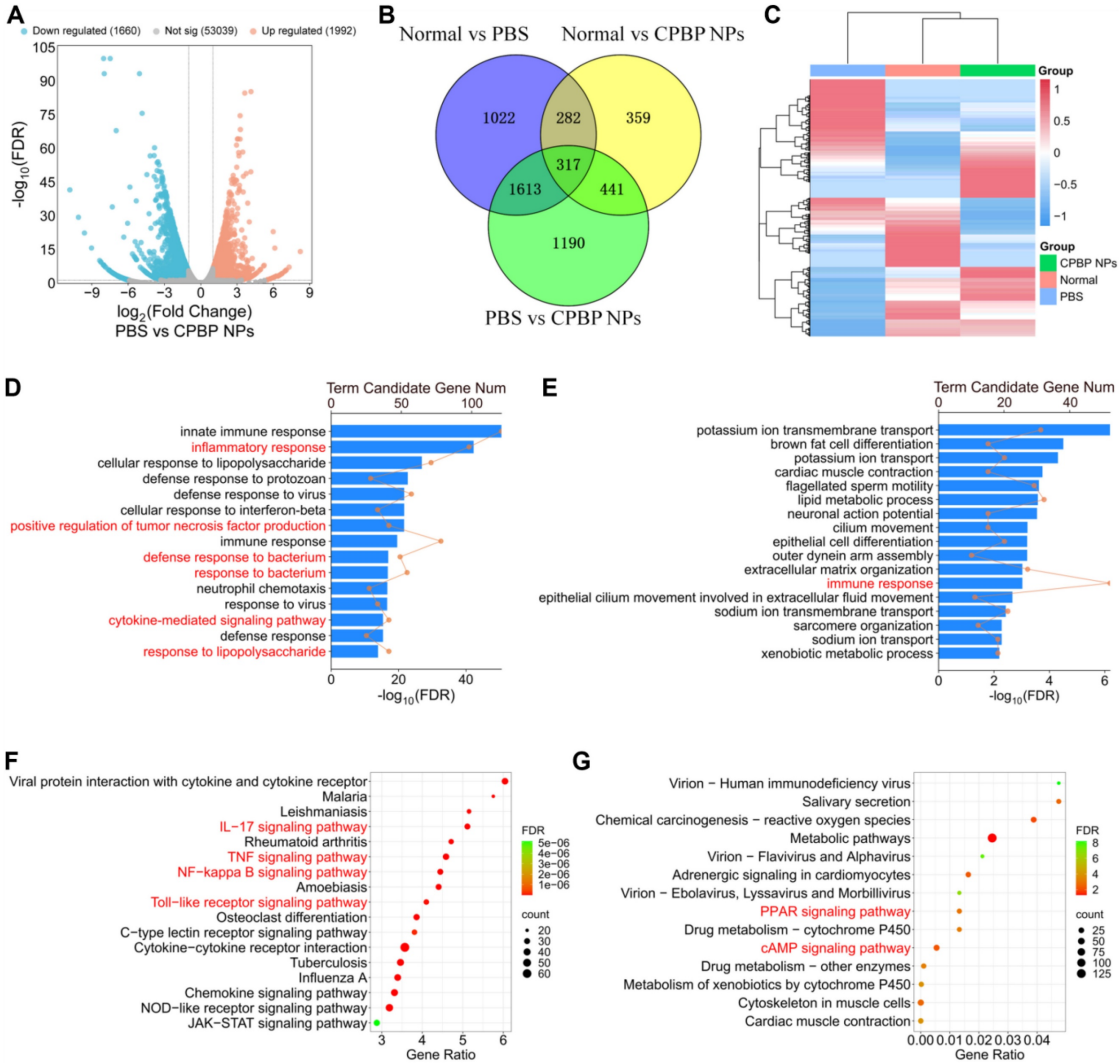
Transcriptomic analysis illustrating the therapeutic mechanism of CPBP NPs in bacterial pneumonia model infected by *S. aureus*. (A) Volcano plot showing DEGs in PBS and CPBP NPs groups. (B) Venn diagrams illustrating the number of DEGs in Normal versus PBS, Normal versus CPBP NPs, and PBS versus CPBP NPs. (C) Heatmap of hierarchical clustering of DEGs. GO enrichment analysis of the gene functions of (D) downregulated and (E) upregulated DEGs. (F) Downregulated and G) upregulated DEGs enriched in the KEGG pathway.

To elucidate the mechanistic underpinnings of CPBP NPs in lung infection protection, we performed a bulk RNA sequencing (RNA-seq) analysis on lung tissues from mice treated in three groups: normal group, PBS group, and CPBP NPs group. Compared to the PBS group, we identified 3652 differentially expressed genes (DEGs) in the CPBP NPs group, with 1660 genes of them being downregulated and 1992 genes being upregulated (**Figure 5A**). We next examined the overlap of DEGs among the normal, PBS and CPBP NPs groups and found 317 coregulated genes (**Figure 5B**). Heatmap cluster analysis of gene expression levels revealed that the CPBP NPs group exhibited a comparable gene expression pattern to the normal group. Conversely, the gene expression levels in both CPBP NPs and normal groups were significantly different from the PBS group (**Figure 5C**). These results suggest that the lung infection in mice treated with CPBP NPs gradually returned to a normal condition.

To clarify the therapeutic mechanisms of CPBP NPs, we performed clustering and enrichment analyses on the DEGs identified between the PBS and CPBP NPs groups. Two widely used analysis methods, Gene Ontology (GO) and Kyoto Encyclopedia of Genes and Genomes (KEGG), were employed to analyze the functions of DEGs between the PBS and CPBP NPs groups. The GO enrichment analysis of biological processes showed that the downregulated DEGs were enriched in pathways related to inflammatory and bacterium response. Specifically, compared to the PBS group, the DEGs in CPBP NPs group were associated with inflammatory response, positive regulation of tumor necrosis factor production, defense response to bacterium, response to bacterium, cytokine-mediated signaling pathway, and response to lipopolysaccharide (**Figure 5D**). Meanwhile, the upregulated DEGs were related to the function of immune response (**Figure 5E**). Additionally, we identified the major pathways of DEGs between the PBS and CPBP NPs groups using KEGG database. The downregulated DEGs were enriched in pro-inflammatory pathways, such as IL-17 signaling pathway, TNF signaling pathway, NF-kappa B signaling pathway, and Toll-like receptor signaling pathway (**Figure 5F**). These pro-inflammatory pathways have been demonstrated to be involved in inflammatory responses during bacterial pneumonia 39-41. As a pro-inflammatory cytokine, IL-17 can synergistically activate the NF-κB pathway together with TNF-α, thereby promoting inflammatory responses 42. Our KEGG analysis results confirmed the involvement of these pathways after CPBP NPs treatment, which was consistent with these previous studies. In contrast, the upregulated DEGs were associated with anti-inflammatory pathways, including PPAR-γ signaling pathway and cAMP signaling pathway (**Figure 5G**). These findings suggest that the CPBP NPs exert a protective effect against lung infection by modulating both anti-inflammatory and antibacterial pathways.

### Antibacterial activity of CPBP NPs in skin wound infection

**Figure 6:**
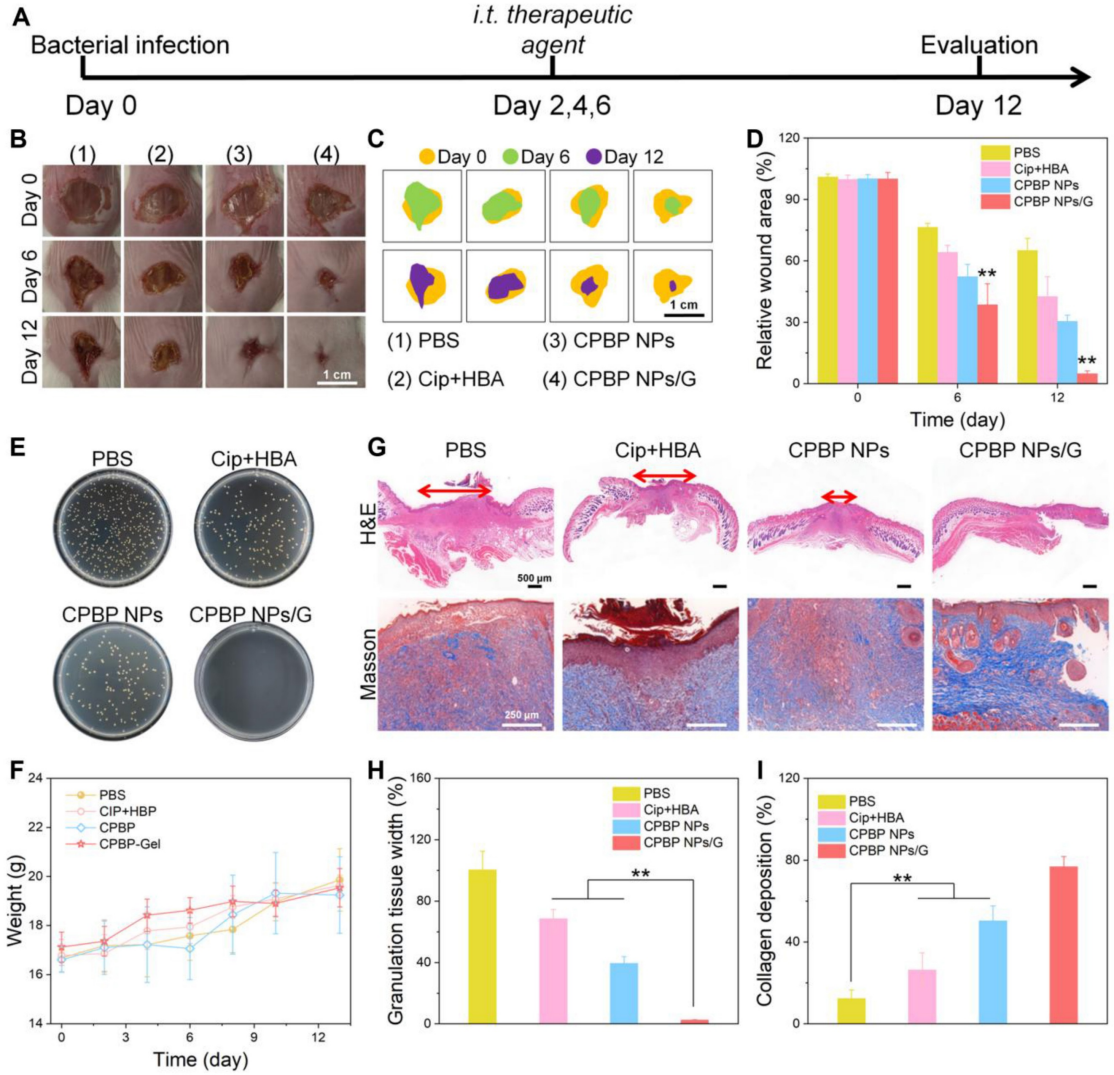
Therapeutic effect on wound healing with *S. aureus*. (A) Schematic diagram of wound healing model treatment schedule, the female BALB/c mice were created 5 mm wounds on the back, after 24 h, subcutaneously injected *S. aureus* suspension (50 μL, 3 × 10^7^ CFU/mL), and infected mice were treated with PBS, Cip+HBA, CPBP NPs, and CPBP NPs/G. (B) Representative photographs of infected wounds on days 0, 6, and 12. (C) Monitoring of wound closure during treatments in each group. (D) The relative wound area in different groups (n = 5), **P < 0.01. (E) Representative photographs of bacterial colonies in *S. aureus*-infected wounds. (F) Corresponding changes in body weight of the mice. (G) H&E and Masson staining images of the infected wound tissues of mice after 12 days of different treatments. Quantitative analysis of stained images acquired from different groups: (H) scar width and (I) college deposition. (n = 5), **P < 0.01.

To further assess the antibacterial efficacy of CPBP NPs in wound healing, wound models measuring 5 mm ~1 cm across were initially established on the dorsal area of female BALB/c mice. Subsequently, *S. aureus* suspension (50 μL, 3 × 10^7^ CFU/mL) was subcutaneously injected into the wound site, as illustrated in **Figure 6A**. After a 24-hour incubation of bacteria beneath the skin, noticeable skin damage was observed at the injection site. The infected mice were then arbitrarily split into four cohorts, each containing eight mice. The cohorts received treatments of PBS, Cip+HBA, CPBP NPs, and fibrin hydrogels loaded with CPBP NPs (designated as CPBP NPs/G), respectively. Representative images of the wound, taken on days 0, 6, and 12, are shown in **Figure 6B, 6C**. On the initial day of injury and infection (day 0), all wounds displayed signs of suppuration, confirming the successful establishment of the wound infection models. By day 12, the wound area in the PBS cohort, Cip+HBA cohort, CPBP NPs cohort, and CPBP NPs/G cohort had reduced to 65.1%, 42.7%, 30.5%, and 4.9%, respectively (**Figure 6D**). To validate the antimicrobial efficacy of various treatments *in vivo*, wound fluid was harvested for bacterial quantification, with colony formation assays conducted on day 12. A notable decrease in bacterial colonies was observed in the CPBP NPs/G cohort (**Figure 6E**). These findings suggest that CPBP NPs/G effectively inhibits bacterial growth on the wound surface, thereby facilitating rapid and efficient skin wound healing. Furthermore, throughout the experiment (from day 0 to day 12), no notable change in the body weight of mice in the CPBP NPs/G treatment cohort was recorded, in contrast to the control cohorts (**Figure 6F**).

To further assess the tissue healing process of wounds, pathological analyses of the wound tissue were conducted using H&E staining. As illustrated in **Figure 6G and H**, the results revealed significant epidermal damage in the PBS and Cip+HBA treatment cohorts. In contrast, after treatment with CPBP NPs and CPBP NPs/G, the formation of numerous blood vessels, hair follicles, and intact epithelial structures were observed. Furthermore, collagen deposition is recognised as pivotal in fostering cell proliferation and facilitating wound healing 43. Masson trichrome staining demonstrated that in the CPBP NPs and CPBP NPs/G treatment cohort, collagen fibres were aligned parallel to the hair follicles, indicating a highly organised and consistent deposition pattern (**Figure 6I**). These findings suggest that CPBP NPs markedly enhance the wound healing process.

**Figure 7:**
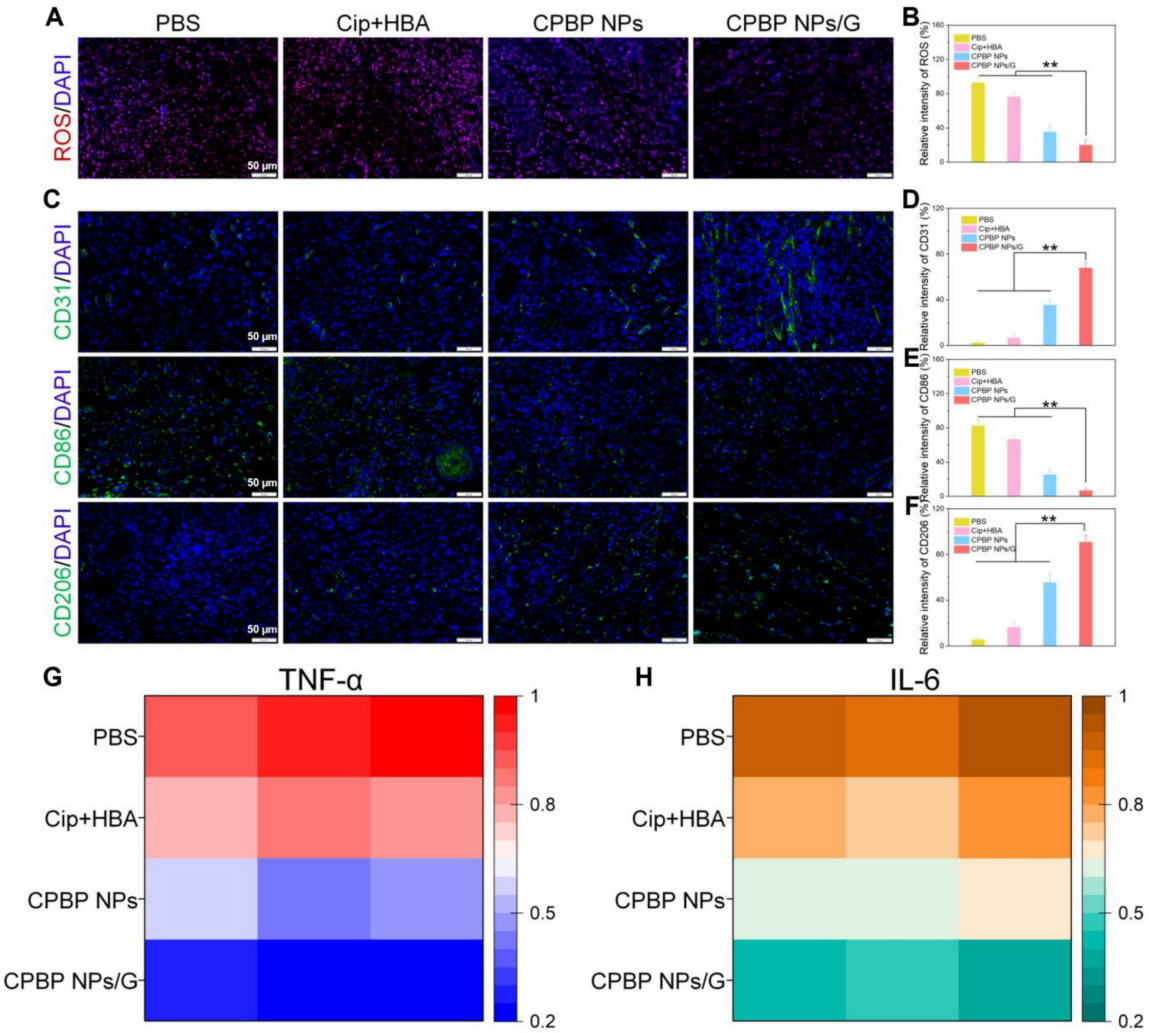
Representative immunofluorescence images (A) and quantitative analysis (B) of ROS expression in infected wounds (n = 5), red is a false color. Representative immunofluorescence images of (C) CD206, CD86, and (D-E) their quantifications of vessel density (n = 5), **P < 0.01. Representative immunofluorescence images of (F) angiogenesis (CD31), and (G) their quantifications of vessel density (n = 5). Quantitative analysis of inflammatory cytokines at the site of infection including TNF-α (G) and IL-6 (H) after 6-days treatment in infected wounds of mice after different treatment groups (n = 5).

Subsequently, DCFH-DA staining was employed as a fluorescent probe to investigate ROS generation *in vivo*. The weakest red fluorescence signal was detected in mice that received intratracheal instillation of CPBP NPs and CPBP NPs/G, in contrast to the PBS and Cip+HBA cohorts, which could be explained by the enzymatic transformation of endogenous H₂O₂ (**Figure 7A, B**).

The evaluation of wound inflammation on day 12 post-treatment involved an examination of the inflammatory status, including macrophage polarisation and the production of inflammatory cytokines within the wound bed. This investigation utilised immunofluorescence staining specific to CD86, CD206, and 4', 6-diamino-2-phenylindole. As depicted in **Figure 7C-E**, the CPBP NPs/G cohorts exhibited reduced CD86 levels alongside elevated CD206 presence, suggesting that CPBP NPs administration promoted greater M2 macrophage polarisation whilst diminishing inflammatory responses. Considering that neovascularisation plays an essential part in delivering oxygen and nutrients vital for tissue restoration, it maintains a fundamental role in wound repair and tissue regeneration during the proliferative stage. Subsequently, the extent of neovascularisation within the granulation tissue underwent evaluation through immunostaining for platelet endothelial cell adhesion molecule-1 (CD31). Compared to the PBS, Cip+HBA, CPBP NPs, and CPBP NPs/G cohorts, the CPBP NPs/G cohort showed elevated CD31 expression on the wound surface (**Figure 7F and 7G**). Collectively, these results suggest that CPBP NPs/G exerts synergistic antibacterial effects via the release of Cip and HBA, thereby effectively inhibiting bacterial growth, accelerating collagen deposition at the wound site, and ultimately facilitating wound healing. To assess the anti-inflammatory effects of CPBP NPs, the pro-inflammatory cytokines TNF-α and IL-6 at the infection site were quantified via ELISA. As shown in **Figure 7H** and **7I**, the expression levels of TNF-α and IL-6 from the infected mice receiving the treatment of CPBP NPs/G was significantly lower than those in other experimental groups, indicating that CPBP NPs could effectively neutralize bacterial toxins and reduce inflammation.

### Biological safety of CPBP NPs

Cytotoxicity and hemolysis serve as critical indicators for evaluating the biocompatibility of drugs. Consequently, the cytotoxicity of CPBP NPs was first assessed using mouse RAW264.7, L-929, and MLE-12 cell lines. As depicted in **Figure S13**, the viability of all cell types maintained above 90% even when exposed to concentrations reaching 200 μg/mL for 24 h, indicating that CPBP NPs exhibit favourable cytocompatibility. Furthermore, a hemolysis test was conducted using fresh red blood cells (RBCs). The hemolytic activity of CPBP NPs was evaluated by observing RBC destruction. As indicated in the inset of **Figure S14**, the supernatant of the water cohort appeared bright red, confirming significant RBC damage. In contrast, the supernatants of the PBS and CPBP NPs (200 μg/mL) cohorts were clear, suggesting no RBC lysis, with the hemolysis ratio of CPBP NPs recorded at only 1.9%. These findings collectively demonstrate that CPBP NPs possess excellent biological safety.

To further evaluate the *in vivo* toxicity of the designed NPs, two cohorts of healthy mice were arbitrarily assigned and treated with either PBS or CPBP NPs via tail vein injection every other day. On day ten, blood biochemistry analyses were conducted, revealing that the liver function markers (aspartate aminotransferase (AST) and alanine aminotransferase (ALT)) and kidney function markers (uric acid (UA), creatinine (CREA) and blood urea nitrogen (BUN)) in the CPBP NPs-treated cohort remained within the normal range, indicating the absence of significant hepatorenal toxicity (**Figure S15**) 44. H&E staining was performed to evaluate pathological alterations in principal organs, further investigating the potential toxicity of CPBP NPs. Furthermore, no inflammatory lesions, histological abnormalities, fibrosis, or necrosis were observed in any of the six cohorts (**Figure S16**). Collectively, these findings confirm the favourable biocompatibility of CPBP NPs, with no significant adverse effects on major organs or blood parameters.

## Conclusion

In conclusion, a self-assembled, H_2_O_2_-activatable hybrid nanoprodrug (CPBP NPs) was successfully developed, integrating the beneficial characteristics of both Cip and boronic acid subunits. CPBP molecule was synthesised through the simple conjugation of boronic acid to Cip, forming colloidally stable nanoassemblies (CPBP NPs) under aqueous conditions. In addition to its ability to scavenge H_2_O_2_, CPBP NPs exhibited potent antioxidant, anti-inflammatory, and anti-apoptotic activities in stimulated RAW 264.7, L-929 and MLE-12 cells. Furthermore, CPBP NPs demonstrated efficient antibacterial effects against *E. coli* and *S. aureus*. In the murine lung infection model, CPBP NPs were preferentially accumulated in the lungs, effectively protecting the lung tissue from bacterial infection by eradicating pathogenic bacteria, suppressing ROS production, and inhibiting the expression of pro-inflammatory mediators, including TNF-α, IL-1β, and IL-6. Moreover, CPBP NPs-loaded fibrin hydrogels (CPBP NPs/G) were shown to promote skin wound healing in mice. These findings collectively suggest that the self-assembled, H_2_O_2_-activatable hybrid nanoprodrug CPBP NPs are promising as novel nanodrugs for treating bacterial infectious diseases. However, in order to promote its clinical conversion, we need to further study its large-up production and long-term safety *in vivo*.

## Supplementary Material

Supplementary materials and methods, figures.

## References

[1] Wang W, Cui Y, Wei X, Zang Y, Chen X, Cheng L (2024). CuCo_2_O_4_ Nanoflowers with multiple enzyme activities for treating bacterium-infected wounds via cuproptosis-like death. ACS Nano.

[2] Finbloom AJ, Raghavan P, Kwon M, Kharbikar NB, Yu AM, Desai AT (2023). Codelivery of synergistic antimicrobials with polyelectrolyte nanocomplexes to treat bacterial biofilms and lung infections. Sci Adv.

[3] Hatlen JT, Miller GL (2021). Staphylococcal Skin and Soft Tissue Infections. Infect Dis Clin North Am.

[4] Roope SJL, Smith DR, Pouwels BK, Buchanan J, Abel L, Eibich P (2019). The challenge of antimicrobial resistance: What economics can contribute. Science.

[5] GBD 2021 Antimicrobial Resistance Collaborators (2024). Global burden of bacterial antimicrobial resistance 1990-2021: a systematic analysis with forecasts to 2050. Lancet.

[6] Yuan H, Hong X, Ma H, Fu C, Guan Y, Huang W (2023). MXene-based dual functional nanocomposite with photothermal nanozyme catalytic activity to fight bacterial infections. ACS Mater Lett.

[7] Wei G, Yang G, Wang Y, Jiang H, Fu Y, Yue G (2020). Phototherapy-based combination strategies for bacterial infection treatment. Theranostics.

[8] Guo L, Tang Y, Wang L, Zhou R, Wang S, Xu H (2024). Synergetic antibacterial nanoparticles with broad-spectrum for wound healing and lung infection therapy. Adv Funct Mater.

[9] Lavergne M, Schaerer R, Grandis De S, Bouheraoua S, Adenuga O, Muralt T (2025). Executioner caspases degrade essential mediators of pathogen-host interactions to inhibit growth of intracellular Listeria monocytogenes. Cell Death Dis.

[10] Vichare R, Kulahci Y, McCallin R, Zor F, Selek FN, Liu L (2025). Theranostic nanoemulsions suppress macrophage-mediated acute inflammation in rats. J Nanobiotechnol.

[11] Yin Z, Song R, Yu T, Fu Y, Ding Y, Nie H (2025). Natural compounds regulate macrophage polarization and alleviate inflammation against ALI/ARDS. Biomolecules.

[12] Retter A, Singer M, Annane D (2025). “The NET effect”: Neutrophil extracellular traps-a potential key component of the dysregulated host immune response in sepsis. Crit Care.

[13] Huang Y, Guo X, Wu Y, Chen X, Feng L, Xie N (2024). Nanotechnology's frontier in combatting infectious and inflammatory diseases: prevention and treatment. Sig Transduct Target Ther.

[14] Zhou J, Rao L, Yu G, Cook TR, Chen X, Huang F (2021). Supramolecular cancer nanotheranostics. Chem Soc Rev.

[15] Yang K, Yang Z, Yu G, Nie Z, Wang R, Chen X (2022). Polyprodrug nanomedicines: An emerging paradigm for cancer therapy. Adv Mater.

[16] Liu Q, Zou J, Chen Z, He W, Wu W (2023). Current research trends of nanomedicines. Acta Pharm Sin B.

[17] Ding Y, Hu X, Piao Y, Huang R, Xie L, Yan X (2023). Lipid prodrug nanoassemblies via dynamic covalent boronates. ACS Nano.

[18] Rautio J, Meanwell NA, Di L, Hageman MJ (2018). The expanding role of prodrugs in contemporary drug design and development. Nat Rev Drug Discovery.

[19] Zhang W, Du X-F, Liu B, Li C, Long J, Zhao M-X (2022). Engineering supramolecular nanomedicine for targeted near infrared-triggered mitochondrial dysfunction to potentiate cisplatin for efficient chemophototherapy. ACS Nano.

[20] Xiang J, Liu X, Yuan G, Zhang R, Zhou Q, Xie T (2021). Nanomedicine from amphiphilizedprodrugs: Concept and clinical translation. Adv Drug Delivery Rev.

[21] Ding C, Chen C, Zeng X, Chen H, Zhao Y (2022). Emerging strategies in stimuli-responsive prodrug nanosystems for cancer therapy. ACS Nano.

[22] Jin L, Zhu Z, Hong L, Qian Z, Wang F, Mao Z (2023). ROS-responsive 18β-glycyrrhetic acid-conjugated polymeric nanoparticles mediate neuroprotection in ischemic stroke through HMGB1 inhibition and microglia polarization regulation. Bioact Mater.

[23] Tian Y, Tang G, Gao Y, Chen X, Zhou Z, Li Y (2022). Carrier-free small molecular self-assembly based on berberine and curcumin incorporated in submicron particles for improving antimicrobial activity. ACS Appl Mater Interfaces.

[24] Jung E, Song N, Lee Y, Kwon G, Kwon S, Lee D (2022). H_2_O_2_-activatable hybrid prodrug nanoassemblies as a pure nanodrug for hepatic ischemia/reperfusion injury. Biomaterials.

[25] Xu Y, Chen Z, Hao W, Yang Z, Farag M, Vong CT (2024). Berberine and magnolol exert cooperative effects on ulcerative colitis in mice by self-assembling into carrier-free nanostructures. J Nanobiotechnology.

[26] Hu Y, Miao Y, Zhang Y, Wang X, Liu X, Zhang W (2024). Co-assembled binary polyphenol natural products for the prevention and treatment of radiation-induced skin injury. ACS Nano.

[27] Li G, Sun B, Li Y, Luo C, He Z, Sun J (2021). Small-molecule prodrug nanoassemblies: An emerging nanoplatform for anticancer drug delivery. Small.

[28] Li M, Zhao L, Zhang T, Shu Y, He Z, Ma Y (2019). Redox-sensitive prodrug nanoassemblies based on linoleic acid-modified docetaxel to resist breast cancers. Acta Pharm Sin B.

[29] Tanita K, Koseki Y, Kamishima T, Kasai H (2020). Tropone skeleton enhances the dispersion stability of nano-prodrugs. Chem Lett.

[30] Xie S, Manuguri S, Proietti G, Romson J, Fu Y, Inge AK (2017). Design and synthesis of theranostic antibiotic nanodrugs that display enhanced antibacterial activity and luminescence. Proc Natl Acad Sci. USA.

[31] Liu H, Tang L, Yin Y, Cao Y, Fu C, Feng J (2024). Photoresponsive multirole nanoweapon camouflaged by hybrid cell membrane vesicles for efficient antibacterial therapy of pseudomonas aeruginosa-infected pneumonia and wound. Adv Sci.

[32] Zhang Q, Jiang Y, Zhang X, Wang Y, Ju R, Wei G (2024). Injectable and near-infrared light-controllable fibrin hydrogels with antimicrobial and immunomodulating properties for infected wound healing. Biomater Res.

[33] Hong S, Lee Y, Shin H, Kim T, Jung E, Lee D (2021). Nanoassemblies of disulfide-bridged bile acid dimers as therapeutics agents for hepatic ischemia/reperfusion injury. ACS Appl Bio Mater.

[34] Lee J, Jeong L, Jung E, Ko C, Seon S, Noh J (2019). Thrombus targeting aspirin particles for near infrared imaging and on-demand therapy of thrombotic vascular diseases. J Controlled Release.

[35] Dhiman BS, Kamat PJ, Naik BD (2009). Antioxidant activity and free radical scavenging reactions of hydroxybenzyl alcohols. Biochemical and pulse radiolysis studies. Chem Biol Interact.

[36] Li Y, Xiu W, Yang K, Wen Q, Yuwen L, Luo Z (2021). A multifunctional Fenton nanoagent for microenvironment-selective anti-biofilm and anti-inflammatory therapy. Mater Horiz.

[37] Nasser A, Dallal MMS, Jahanbakhshi S, Azimi T, Nikouei L (2022). *Staphylococcus aureus*: biofilm formation and strategies against it. Curr Pharm Biotechnol.

[38] Leng T, Zhang L, Ma J, Qu X, Lei B (2024). Intrinsically bioactive multifunctional Poly(citrate-curcumin) for rapid lung injury and MRSA infection therapy. Bioact Mater.

[39] Jones MR, Simms BT, Lupa MM, Kogan MS, Mizgerd JP (2005). Lung NF-kappa B activation and neutrophil recruitment require IL-1 and TNF receptor signaling during pneumococcal pneumonia. J Immunol.

[40] Mizgerd JP, Peschon JJ, Doerschuk CM (2000). Roles of tumor necrosis factor receptor signaling during murine Escherichia coli pneumonia. Am J Respir Cell Mol Biol.

[41] Ye P, Rodriguez FH, Kanaly S, Stocking KL, Schurr J, Schwarzenberger P (2001). Requirement of interleukin 17 receptor signaling for lung CXC chemokine and granulocyte colony-stimulating factor expression, neutrophil recruitment, and host defense. J Exp Med.

[42] Suyama K, Sakai D, Watanabe M (2022). The role of IL-17-mediated inflammatory processes in the pathogenesis of intervertebral disc degeneration and herniation: A comprehensive Review. Front Cell Dev Biol.

[43] Xiu W, Wan L, Yang K, Li X, Yuwen L, Dong H Potentiating hypoxic microenvironment for antibiotic activation by photodynamic therapy to combat bacterial biofilm infections. Nat Commun. 2022: 13: 3875.

[44] Lu M, Xing H, Shao W, Zhang T, Zhang M, Wang Y (2022). Photoactivatable silencing extracellular vesicle (PASEV) sensitizes cancer immunotherapy. Adv Mater.

