# Supplementary material for: A self-assembled and H2O2-activatable hybrid nanoprodrug for lung infection and wound healing therapy
Source: Theranostics. 2025 Apr 28;15(12):5953–68. doi: 10.7150/thno.114344 (PMC12068296; doi:10.7150/thno.114344)
Supplement: Supplementary file 1 — Supplementary materials and methods, figures. [file thnov15p5953s1.pdf]

Supporting Information

A self-assembled and H_2O_2-activatable hybrid nanoprodrug for lung infection and wound healing therapy

Rui Zhang#, Danhua Mao#, Yiyong Fu#, Rong Ju, and Guoqing Wei*

Chengdu Women's and Children's Central Hospital, School of Medicine, University of Electronic Science and Technology of China, Chengdu, 611731, China

#These authors contributed equally to this work.

*Correspondence should be addressed to Guoqing Wei (guoqing@uestc.edu.cn)

Materials and methods

Characterisation

The molecular structures of the synthesised compounds were verified via ^{1}H nuclear magnetic resonance (NMR) and ^{13}C NMR spectroscopy, conducted at ambient temperature utilising a Varian 400 MHz NMR spectrometer (Agilent Technologies, USA). Ultraviolet-visible (UV–vis) spectra and Fourier transform infrared (FT-IR) spectra were ascertained employing a UV-Vis spectrometer (UV-2550, Shimadzu, Japan) and a Nicolet 5700 spectrometer, respectively. The dimensional characteristics and zeta potential of CPBP NPs were determined through DLS employing a Zetasizer Nano ZS90 (Malvern Instruments, UK). The morphology of CPBP NPs was examined via SEM (Zeiss Merlin FE-SEM). Matrix-assisted laser desorption/ionisation-time of flight MS was performed on an AutoFlex Max Mass Spectrometer (Bruker, Germany). High-performance liquid chromatography (HPLC) analysis was conducted using a 1260 Infinity II High-performance liquid chromatography system (Agilent, USA) equipped with an Alltima C18 column (4.6 × 150 mm, 4 μm).

Synthesis of Intermediate 1

Cip (0.5 g, 1.5 mmol) underwent dissolution in THF (30 mL), whilst Boc_2O (490 mg, 2.25 mmol) was incorporated during ice bath conditions, succeeded by ambient

temperature agitation for 4 h. To the resulting mixture, 100 mL of deionised water was introduced. The precipitate was subsequently filtered and dried under vacuum conditions. Intermediate 1 was isolated as a white powder (530 mg, 81.5% yield). The ^{1}H NMR (400 MHz, DMSO-d_6) spectrum exhibited the following chemical shifts: δ 8.68 (s, 1H), 7.94 (d, J = 13.2 Hz, 1H), 7.60 (d, J = 7.4 Hz, 1H), 3.82 (s, 1H), 3.56 (s, 4H), 3.31 (s, 4H), 1.44 (s, 9H), 1.34 (d, J = 6.8 Hz, 2H), 1.19 (s, 2H).

Synthesis of phenylboronic acid-functionalised ciprofloxacin (CPBP)

Intermediate 1 (500 mg, 1.15 mmol), triethylamine (150 mg, 1.45 mmol), 2,4,6-trichlorobenzoyl chloride (300 mg, 1.27 mmol), and 4-dimethylaminopyridine (DMAP) (170 mg, 1.38 mmol) were combined in 1,4-dioxane (20 mL) and agitated at 25 °C for 15 min. Subsequently, 4-hydroxymethylphenylboronic acid (210 mg, 1.38 mmol) was introduced into the mixture, which was then agitated at 25 °C for a further hour. Following this, the reaction solution was combined with brine (80 mL) and subjected to extraction with ethyl ether (3 × 80 mL). The organic phases were combined, rinsed with brine (2 × 40 mL), dried using sodium sulfate (Na_2SO_4), and concentrated under diminished pressure. Purification through silica gel column chromatography yielded Intermediate 2 as a white solid (380 mg, 70% yield). The ^{1}H NMR (400 MHz, DMSO-d_6) spectrum revealed the following chemical shifts: δ 8.51 (s, 1H), 8.12 (s, 2H), 7.79 (d, J = 7.6 Hz, 2H), 7.44 (dd, J = 14.8, 7.6 Hz, 2H), 5.26 (s, 2H), 4.08 (m, 1H), 3.97 (br, 2H), 3.56 (m, 4H), 3.17 (m, 4H), 1.42 (s, 9H), 1.32 (m, 2H), 1.17 (m, 2H).

Intermediate 2 (380 mg, 0.81 mmol) was solubilised in tetrahydrofuran (THF, 5 mL), and 2 M hydrochloric acid (5 mL, 10 mmol) was subsequently added to the solution. The resulting mixture was stirred at room temperature for 2 h. Following this, the THF solvent was removed under diminished pressure, and the remaining was extracted with ethyl acetate (3 × 80 mL). The merged organic layers were dehydrated with anhydrous sodium sulfate (Na_2SO_4), succeeded by solvent elimination under diminished pressure, furnishing CPBP (262 mg, 83% yield) as a white solid. The ^1H NMR (400 MHz, DMSO-d_6) spectrum exhibited the following chemical shifts: δ 8.49 (s, 1H), 8.07 (s, 1H), 7.80 (d, J = 8.9 Hz, 2H), 7.44 (dd, J = 14.8, 7.6 Hz, 2H), 5.27 (s, 2H), 3.70 (m, 3H), 3.14 (m, 4H), 2.54 (m, 4H), 1.88 (s, 1H), 1.27 – 1.09 (m, 4H).^{13}C NMR (400 MHz, DMSO-d_6) δ 172.53, 172.16, 164.93, 149.05, 138.69, 134.65, 126.94, 122.75, 112.29, 112.14, 109.27, 107.00, 65.71, 47.98, 43.67, 35.39, 21.63, 8.03.

Supplementary Figures

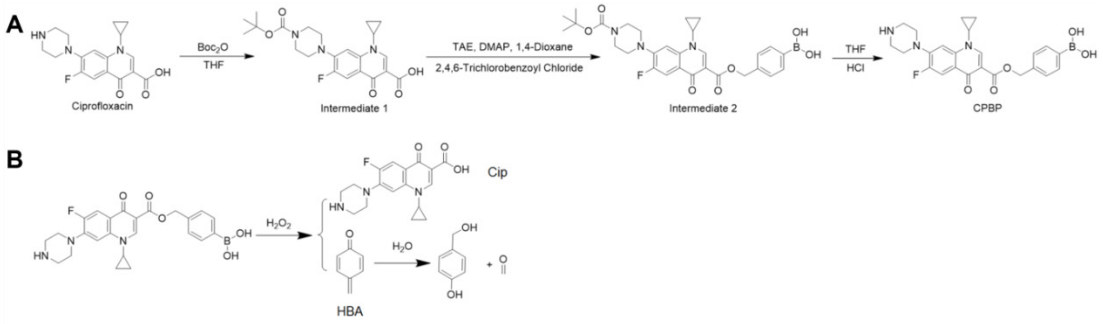

Figure S1. (A) Synthetic route of CPBP molecule. (B) Structural change diagram of CPBP molecule reacting with H_2O_2 aqueous solution.

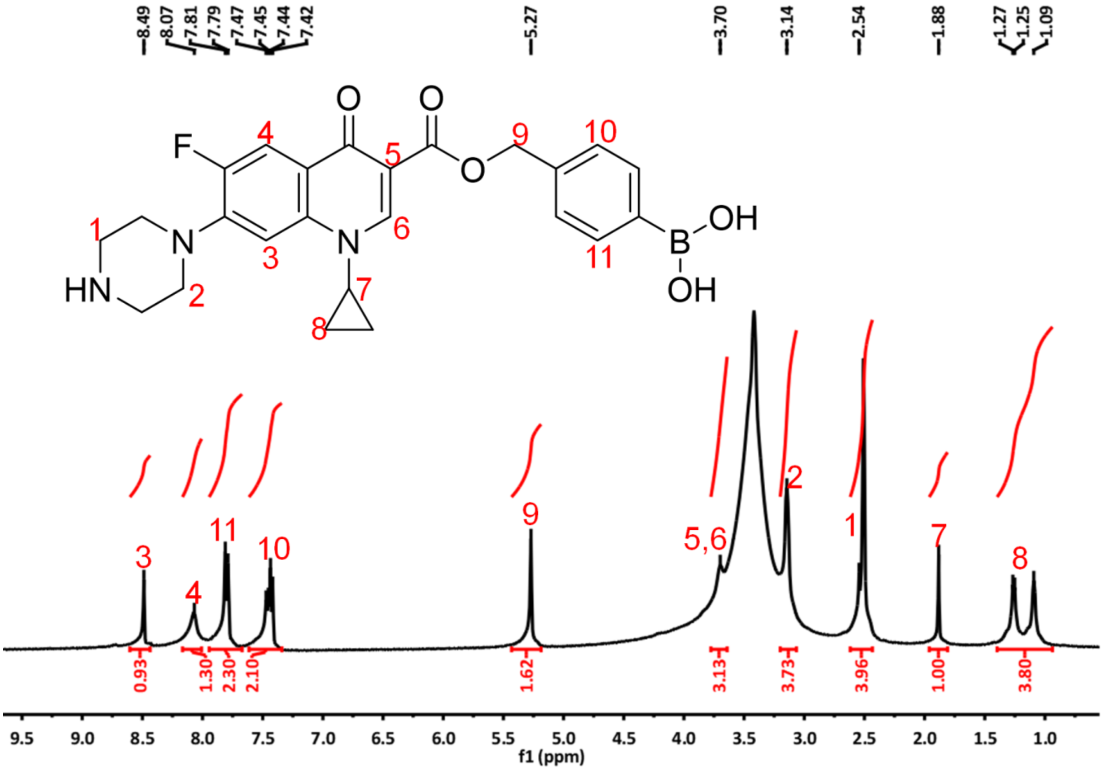

Figure S2. ^{1}H NMR spectrum of CPBP molecule in d_6-DMSO.

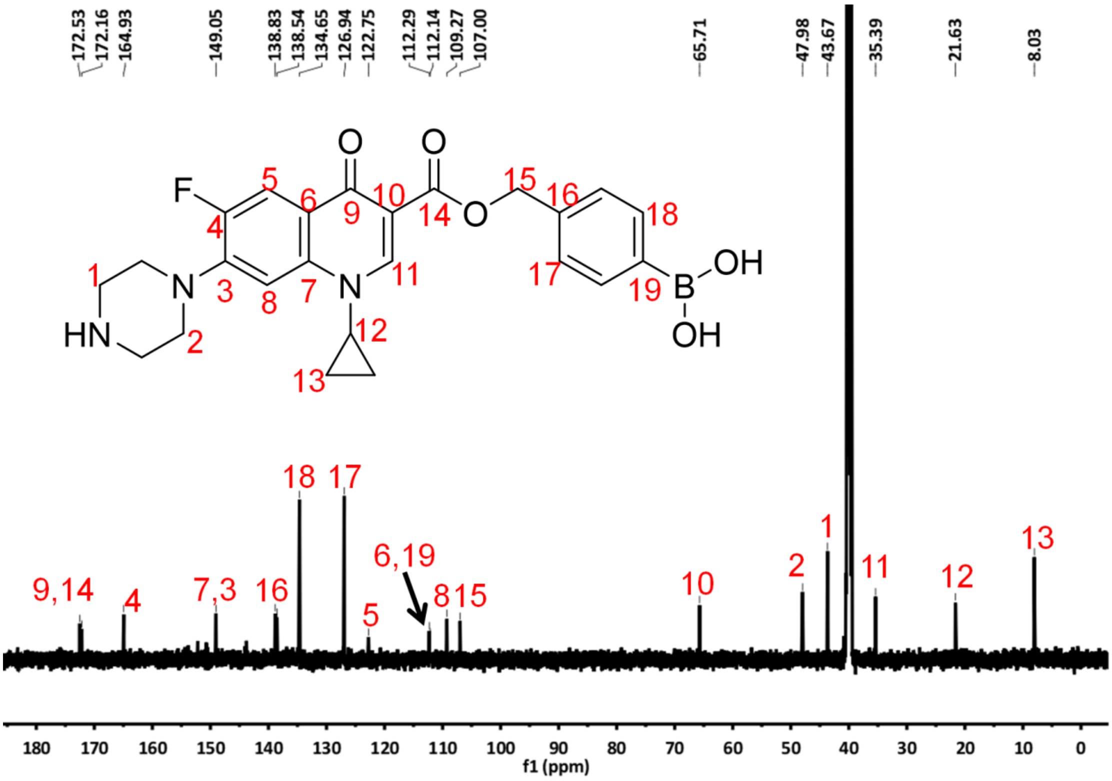

Figure S3. ^{13}C NMR spectrum of CPBP molecule in d_6-DMSO.

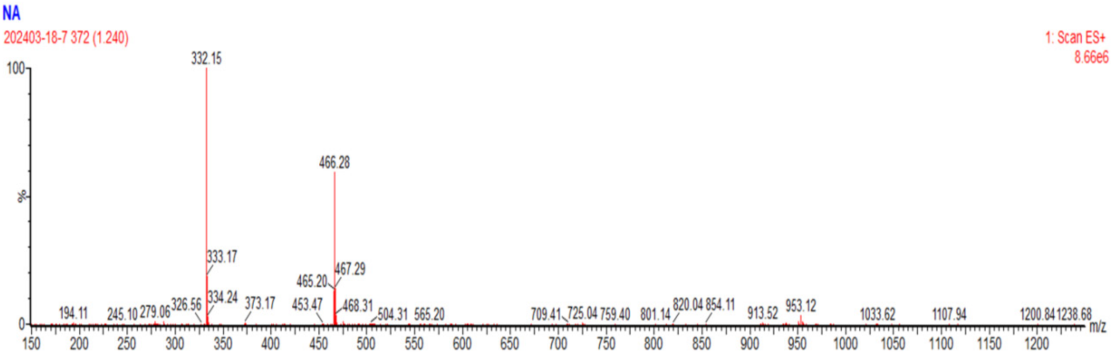

Figure S4. Mass spectrum of CPBP molecule.

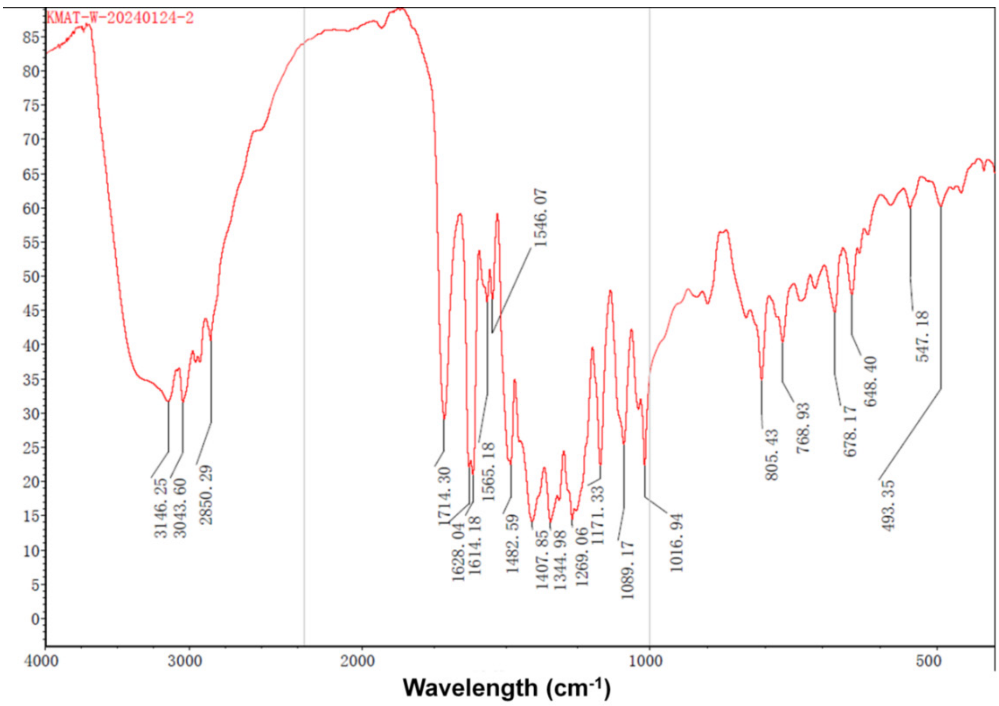

Figure S5. FT-IR spectra of CPBP molecule.

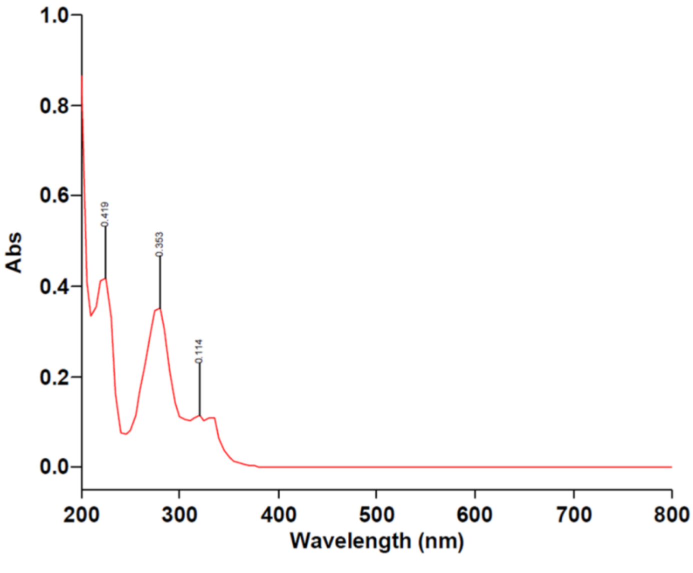

Figure S6. UV–vis absorption spectra of CPBP.

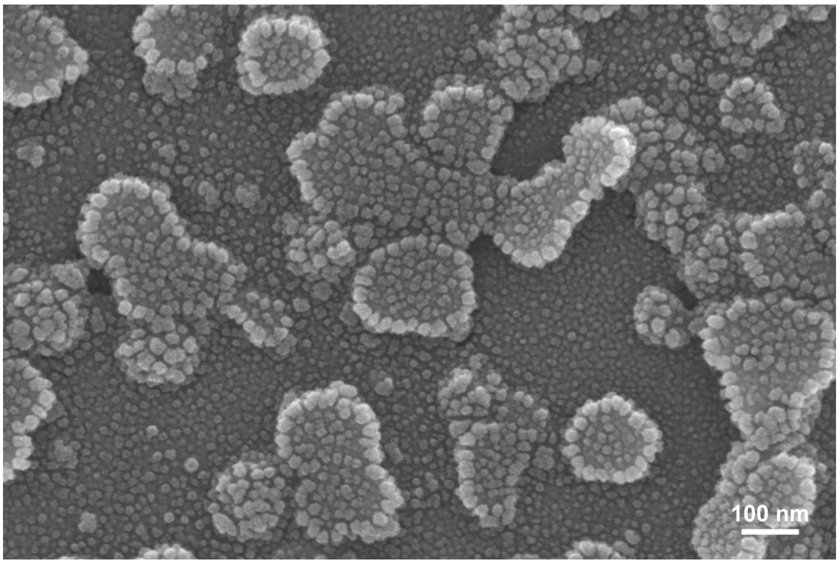

Figure S7. SEM image of CPBP NPs after the H_2O_2 treatment for 12 h.

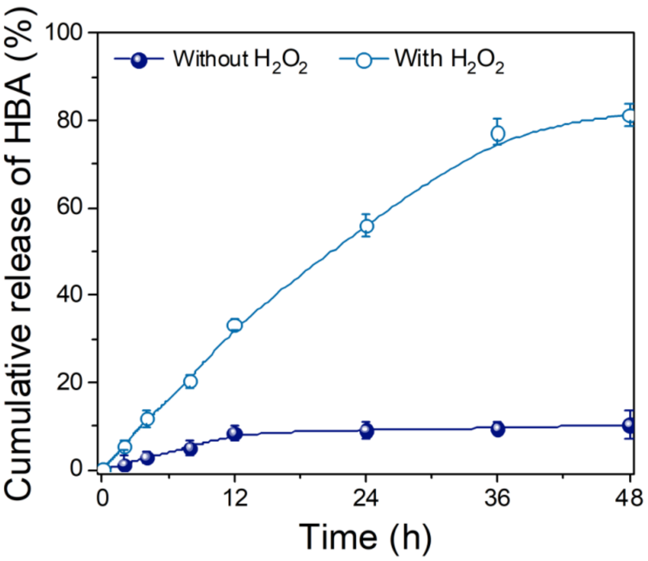

Figure S8. Cumulative release of HBA from CPBP NPs (n=3).

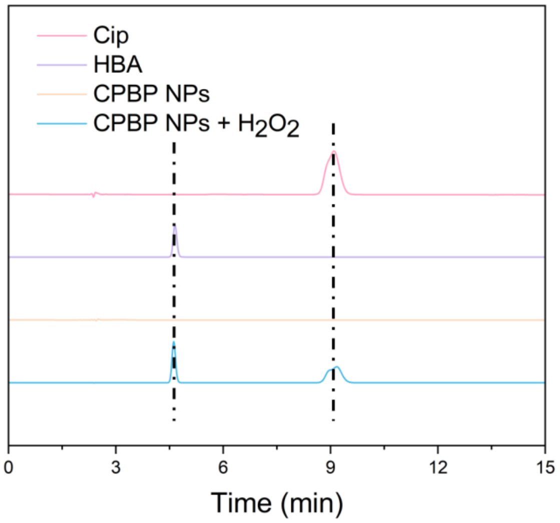

Figure S9. HPLC chromatogram of Cip, HBA and CPBP NPs after the H_2O_2 treatment or not.

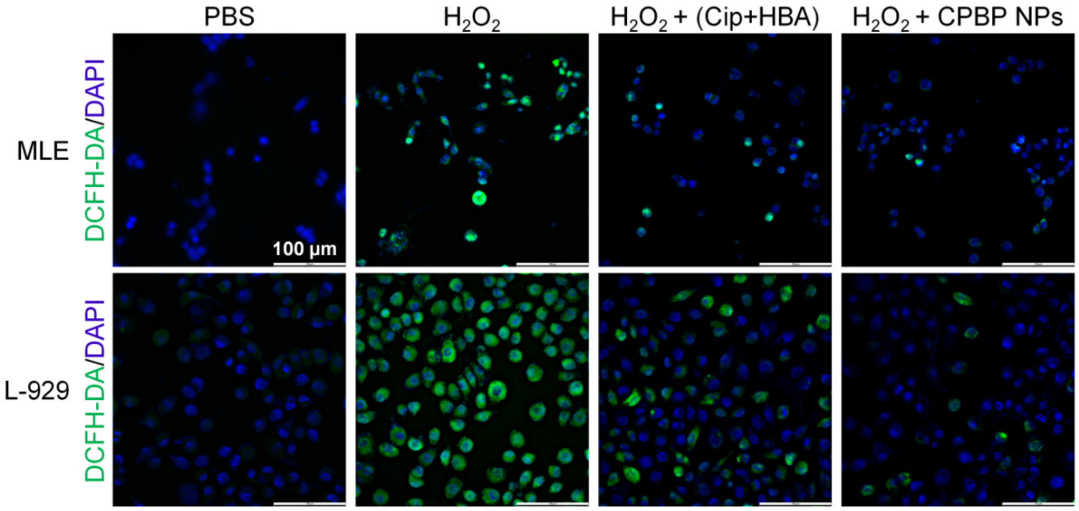

Figure S10. Suppressive effects of Cip+HBA or CPBP NPs on the generation of intracellular ROS in H_2O_2-stimulated MLE-12 and L-929 cells. DAPI: excitation wavelengths is 405 nm, DCFH-DA: excitation wavelengths is 488 nm.

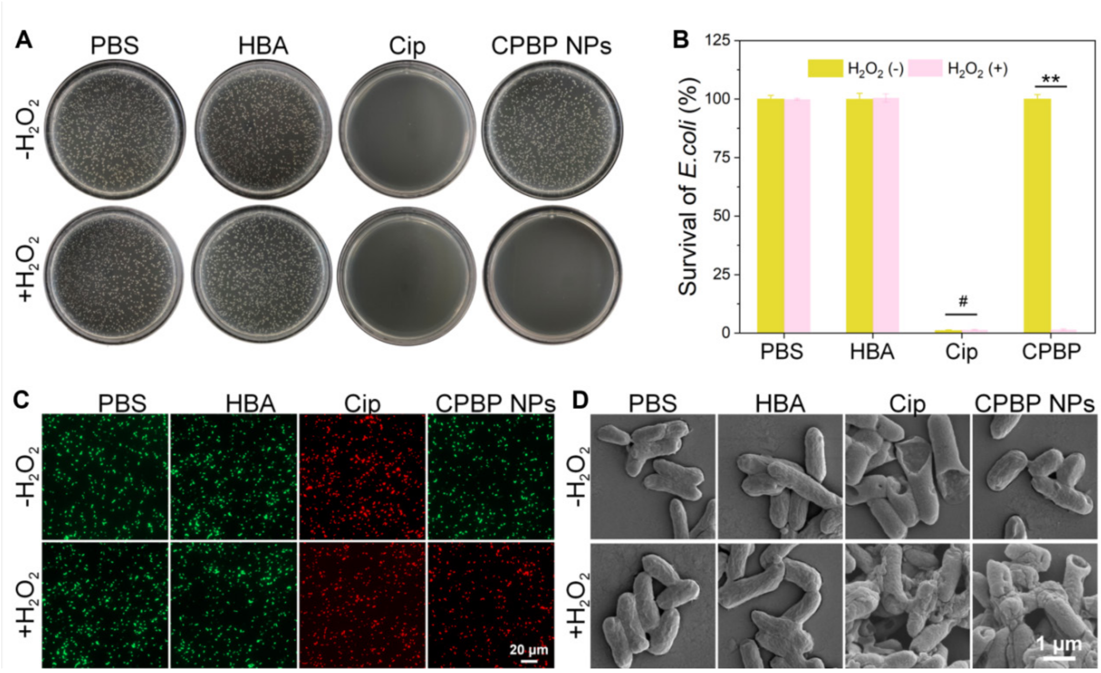

Figure S11. (A) Representative photographs and (B) their quantitative analysis of bacterial colonies formed on agar plates in different treatment groups (n = 3), $^{**}P < 0.01$ and $^{\#}P > 0.05$. (C) Fluorescence images for *E. coil* after various treatments, green and red represent live and dead bacteria respectively. (D) SEM images of *E. coil* after various treatments.

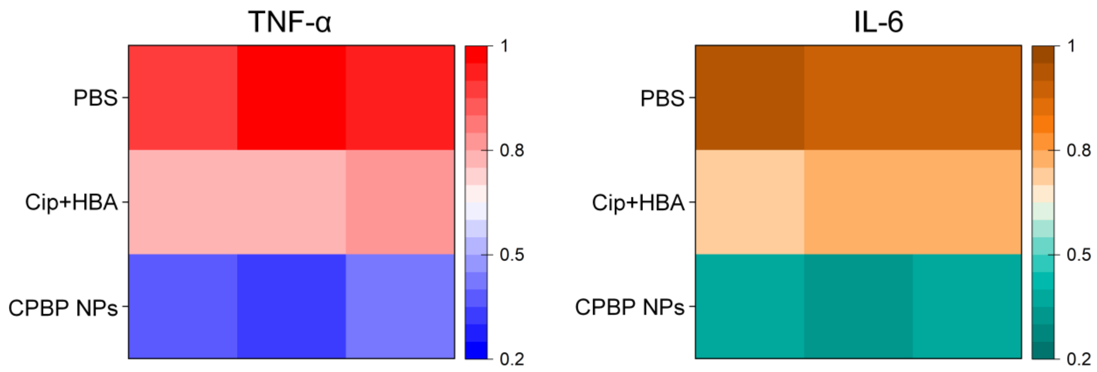

Figure S12. Quantitative analysis of inflammatory cytokines at the site of lungs infection of mice including TNF-αand IL-6 after different treatment (n = 3).

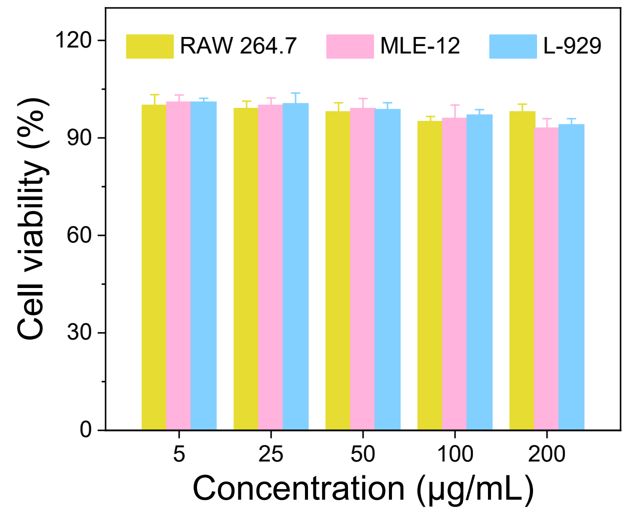

Figure S13. Cell viability of RAW264.7, MLE-12, and L-929 cells treated with different concentrations of CPBP NPs (n = 3).

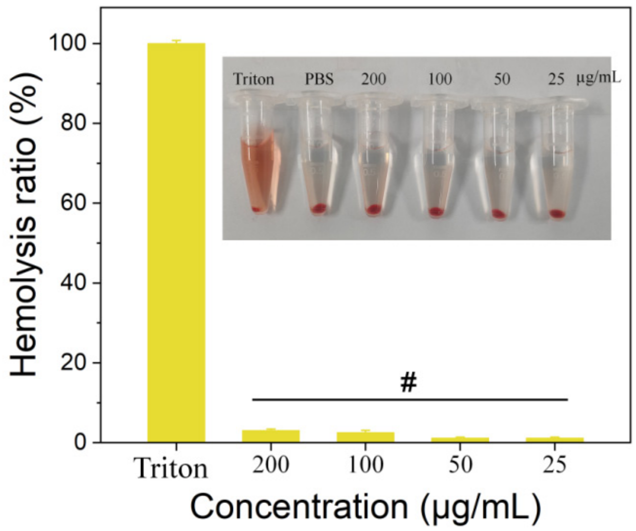

Figure S14. Relative hemolysis ratios of different concentrations of CPBP NPs (n = 3), $^{\#}P > 0.05$, the illustrations are physical photos.

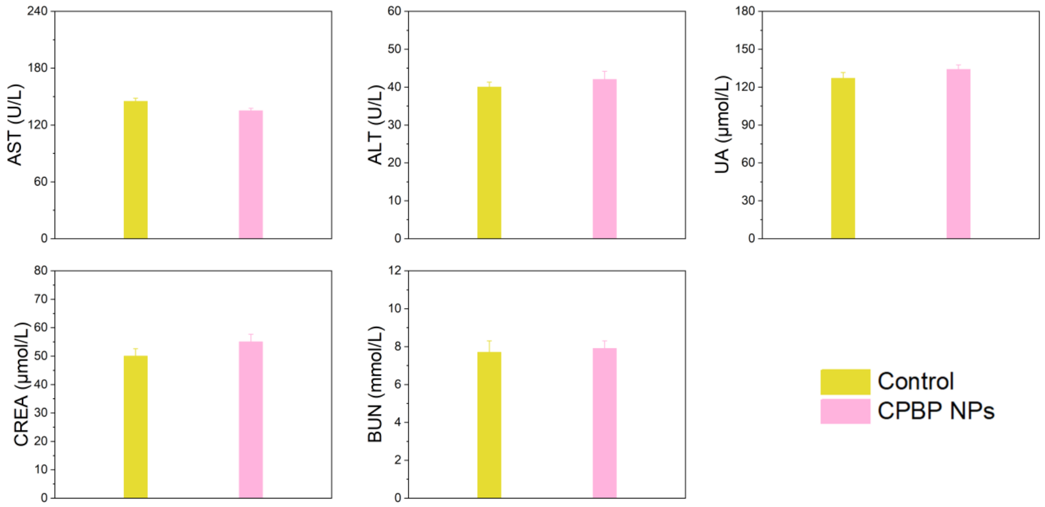

Figure S15. Evaluation of liver and kidney function via biomarker analysis of blood serum (n = 3).

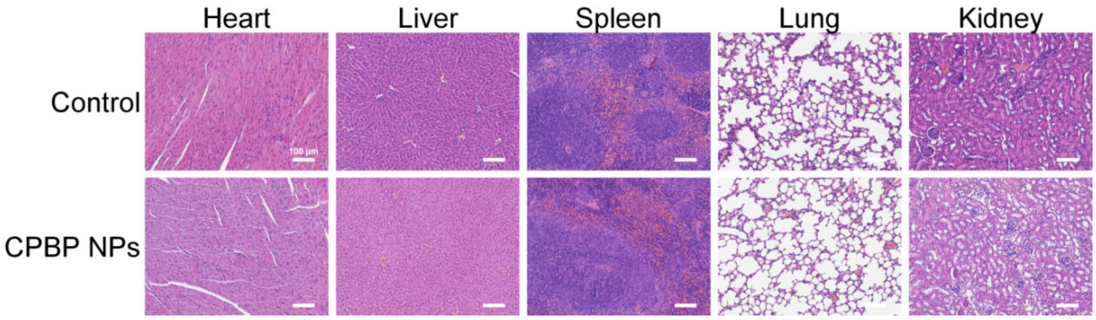

Figure S16. Histological images of heart, liver, spleen, lung, and kidney samples from mice after i.v. injection of CPBP NPs, normal mice was used as control.
